# Decoupling the Roles of Chain Length, Entanglements, and Intermolecular Interactions on the Melt Memory of Semicrystalline Polar Homopolymers

**DOI:** 10.1021/acs.macromol.5c03323

**Published:** 2026-03-07

**Authors:** M. Ali Aboudzadeh, Leire Sangroniz, Olivier Coulembier, Marcello Ferranti, Salvatore Costanzo, Nino Grizzuti, D. Cavallo, Alejandro J. Müller

**Affiliations:** † POLYMAT and Department of Applied Chemistry, Faculty of Chemistry, University of the Basque Country UPV/EHU, Paseo Manuel de Lardizabal 3, Donostia-San Sebastián 20018, Spain; ‡ POLYMAT and Department of Polymers and Advanced Materials: Physics, Chemistry and Technology, Faculty of Chemistry, University of the Basque Country UPV/EHU, Paseo Manuel de Lardizábal, 3, Donostia-San Sebastián 20018, Spain; § Laboratory of Polymeric and Composite Materials, University of Mons, Place du Parc 23, Mons 7000, Belgium; ∥ Department of Chemical, Materials, and Production Engineering (DICMAPI), University of Naples Federico II, P.le Tecchio 80, Naples 80125, Italy; ⊥ Department of Chemistry and Industrial Chemistry, 9302 University of Genoa, Via Dodecaneso 31, Genoa 16146, Italy; # Ikerbasque-Basque Foundation for Science, Plaza Euskadi 5, Bilbao 48009, Spain

## Abstract

In polymer crystals, chains are closely packed within unit cells. If they are heated above their melting point, they require a specific temperature and time to revert their ordered conformations to isotropic random coils in the melt. When the temperature is slightly above the melting point and all crystals have melted, the chains may retain a memory of the conformations they had in the crystals, i.e., they remember some of the extended or oriented conformations that they had in crystallographic registry. This causes enhanced recrystallization, a property denoted melt memory. Its exact nature remains a central question in polymer crystallization. Here, we combine small-angle X-ray scattering (SAXS) and differential scanning calorimetry (DSC) self-nucleation experiments to systematically investigate the molecular origin of melt memory in poly­(ε-caprolactone) (PCL) and poly­(ethylene oxide) (PEO) model samples, spanning a range of molecular weights from oligomers to highly entangled polymers. The entanglement molecular weights (*M*_e_) were experimentally determined with rheological techniques using a large number of samples. To quantify intermolecular interactions and rheological constraints, we introduce a dimensionless interaction index that accounts for crystallinity-weighted intermolecular interactions and chain packing in the melt. This index rises sharply in oligomeric samples and attains a maximum near *M*_e_. Without strong enough intermolecular interactions, melt memory cannot develop; for example, linear polyethylene does not exhibit melt memory. Conversely, in polar homopolymers, there is a critical chain length below which the intermolecular interaction density is not enough for memory to develop. Beyond this minimum chain length, melt memory is observed in polar homopolymers even in the absence of entanglements, in which case it is exclusively due to intermolecular interactions. Beyond *M*_e_, the melt memory increases as entanglements preserve the melt’s complexity, characterized by intermolecular interactions. These results establish a unified structure–property framework that links molecular weight, morphology, and intermolecular interactions to the melt memory of semicrystalline polar homopolymers.

## Graphical abstract

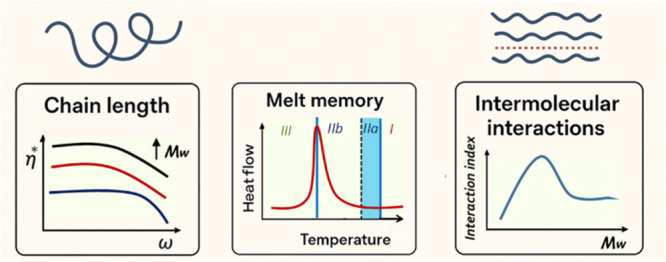

## 1 Introduction

Melt memory is a well-known feature of many semicrystalline polymers, in which remnants of crystalline or molecular order persist after melting and influence subsequent crystallization upon cooling. Such residual order can lower the nucleation energy barrier, act as self-nuclei, and accelerate crystallization rates.^1,2^ This phenomenon, known as “melt memory,” has been widely discussed for decades.^3–9^

In this paper, we specifically focus on the melt memory of PCL and PEO, as quantified by the width of the **melt memory *Domain*** obtained through self-nucleation experiments (i.e., *Domain IIa*), as described in detail in the experimental section. This melt memory ***Domain IIa*** can be achieved by heating a polar, high-molecular-weight, semicrystalline polymer to a temperature just above its end-melting temperature, as determined by DSC. This should be clearly distinguished from the most common self-nucleation case caused by self-seeding (within the **self-seeding** *Domain*, or ***Domain IIb***). Self-seeding can be induced by heating any semicrystalline polymer to a temperature high enough to melt most of the crystals but low enough to leave crystal fragments that can act as ideal epitaxial nucleating seeds upon recrystallization.

PCL is a biodegradable, semicrystalline polyester widely used in biomedical, environmental, and packaging applications due to its low melting point, high flexibility, and ease of processing. PEO is one of the most important polyethers due to its water solubility and biocompatibility. It has many applications in pharmaceuticals and energy storage because of its high chain flexibility, low toxicity, and ability to form complexes with various salts and polar molecules. Both PCL and PEO are linear, flexible semicrystalline polymers with low glass transition temperatures (*T*_g_ ≈ −60 °C for PCL, *T*_g_ ≈ −65 °C for PEO) and high chain mobility. Although these features suggest that an isotropic melt could be readily obtained when these polymers are heated just above their melting peaks, both polymers exhibit significant melt memory in the high-molecular-weight range commonly studied in the literature.^6,10–16^

The exact nature of melt memory remains a topic of debate, despite significant advances in numerous studies. It is now well-known that, for homopolymers, only those with intermolecular interactions stronger than van der Waals forces exhibit melt memory. For instance, linear polyethylene or polypropylene does not exhibit melt memory; only self-seeding occurs. This indicates that among linear homopolymers, only the polar ones exhibit significant melt memory. It has been argued that such strong intermolecular interactions within the well-packed chains in the crystal lattice can persist after melting (depending on the temperature employed and the time this temperature is applied) in regions of residual chain orientation in the melt, which can serve as self-nuclei during subsequent cooling.^6,15,17^ In the case of random copolymers, even polyolefins can display melt memory for different phenomena (segregation of the crystallizable chain segments) that induce complex melt topologies. The reader is referred to the following references for recent works on random copolymer melt memory studies.^8,18–27^

Recent studies on PCL and PEO crystallization indicate that several factors should be considered, as they can determine the origin and strength of melt memory: chain length, intermolecular interactions, and entanglement constraints in the melt.^13–16,28,29^ The term chain length in this manuscript refers to the average chain length of a polymer molecule (i.e., its contour length), which is directly proportional to the chain molar mass. Yu and co-worker demonstrated that in PCL/SAN blends, the lifetime of melt memory far exceeds both segmental orientation and diffusion time scales and instead closely follows re-entanglement kinetics, providing direct evidence that chain re-entanglements underpin melt memory at temperatures well above *T*_m*.*_ ^29^ Similarly, Kurz et al. used rheology, NMR, and SAXS to correlate enhanced lamellar-growth rates and crystallinity with a slowly relaxing “entanglement-rich” zone in the amorphous phase, underscoring how melt-phase entanglements act as persistent memory nuclei.^30^

We recently studied the effects of molecular weight on the melt memory of PEO^16^ using a set of closely monodispersed PEO molecular weight gel permeation chromatography standards. Based on literature reports of the entanglement molecular weight of PEO, we found that melt memory only appeared when entanglements were present in the sample. However, we did not directly measure the entanglement molecular weight for our samples. Fetters et al.^31^ reported a value of *M*_e_ of about 1700 g/mol. Another study^32^ considered the zero shear viscosity as a function of molar mass, observing a deviation from linearity above 1000 g/mol. Therefore, we previously concluded^16^ using literature *M*_e_ values, that PEO melt memory arose predominantly from entanglement constraints, as samples with *M*_w_ lower than 1 kg/mol did not exhibit melt memory.

Apart from entanglement constraints, weak dipole–dipole interactions may also hinder complete randomization of the melt state, even though PCL is not strongly polar. Funaki et al.^33^ employed terahertz and IR spectroscopy combined with quantum-chemical calculations to identify three types of weak C–H···O=C hydrogen bonding in PCL. These interactions explain why PCL displays melt memory despite being only weakly polar.^33^ We have also reported evidence of reduced dielectric permittivity in self-nucleated PCL melts within *Domain IIa* compared to isotropic melts. We attributed this to dipoles being partially “restricted″ by residual segmental interactions in the crystals that survive melting.^34^

Also, for PCL, the extent of the melt memory window has been found to depend on factors such as molecular weight, intermolecular interactions, chain dynamics, thermal history, or cooling and heating rate.^13–16,28,29,35^ Some of us have recently studied the effect of molecular weight (covering a wide range of number-average molecular weights (M_*n*_) between 0.48 and 70.5 kg/mol) on the crystallization behavior and melt memory of PCL.^13^ Our findings demonstrated that increasing PCL’s molecular weight above the entanglement molecular weight (*M*_e_), and thus the number of entanglements, systematically enhances melt memory. In that study, among the tested *M*_n_ *s*, we had only one available sample below the entanglement molecular weight (*M*_e_) reported in the literature for PCL, and this sample did not display any memory. However, this single data point was insufficient to conclusively attribute the origin of melt memory to the presence of entanglements.^13^

Despite these insights, the critical chain length at which melt memory first appears in polar homopolymers remains undefined. For PCL, sub-*M*_e_ data are scarce and inconclusive. For PEO, our previous work^16^ that claimed the crucial importance of entanglements to trigger melt memory was based on a key reference from 1965.^32^ We based our analysis^16^ on literature reports where *M*_e_ was obtained by rheological measurements,^31,32^ not considering NMR results^16,36^ that display higher *M*_e_ values than those obtained with rheological techniques.

Moreover, there is a lack of direct comparisons regarding how molecular weight influences melt memory across polar polymers. This raises two unresolved questions: (i) how far below *M*_e_ melt memory can exist in polar homopolymers, and (ii) how differences in intermolecular or intersegmental interaction strength between PCL and PEO affect this critical chain length threshold for melt memory to develop. Here, we addressed these questions through a systematic study of well-defined, model series of PCL and PEO samples covering molecular weights from the oligomeric regime to well above *M*_e_. Carefully performed rheological experiments were carried out on a broad range of samples to obtain accurate values of *M*_e_ for PCL and PEO. Using a combination of DSC, SAXS, and comprehensive rheological analysis, we establish direct links between chain length, chemical structure, morphology, and melt memory. Specifically, DSC was used to map the melt-memory temperature window as a function of chain length via self-nucleation (SN) experiments, SAXS to measure the thicknesses of the lamellar and amorphous layers, and rheology to determine reliable *M*_e_ values from both steady-shear viscosity and plateau modulus measurements.

To directly capture the role of cohesive energy density (influenced by intermolecular or intersegmental interactions), we introduce an interaction index, defined as the product of Hansen interaction solubility parameters and crystallinity, divided by the square root of the shear modulus, which enables comparison between interaction-dominated and entanglement-reinforced melt memory. Our results show that in both PEO and PCL, melt memory can develop at chain lengths below the experimentally determined entanglement molecular weight *M*_e_. There is a critical chain length that depends on the strength of segmental interactions needed to display melt memory. However, the strength of melt memory (defined as the temperature width of *Domain IIa*) is governed by the combined effects of the density of intermolecular interactions and the number of entanglements present in the polymer. By directly comparing two polar homopolymers with distinct polar groups, ester-based in PCL and ether-based in PEO, this work establishes a unified framework for rationalizing the critical chain length required for melt memory in polar polymers. Finally, we compare the results with linear polyethylene (PE), which has no melt memory, regardless of its molecular weight. PE is also a highly flexible polymer like PEO and PCL, but lacks significant intermolecular interactions (as it only contains weak van der Waals forces between the chains in the crystal lattice), thus we verified that intermolecular interactions are indispensable for melt memory in linear homopolymers, while entanglements are only effective in strengthening melt memory when intermolecular interactions are present. The comprehensive approach employed in this paper, using a large number of model samples in a broad molecular weight range, also enabled us to reassess the entanglement molecular weight of PEO and PCL with unprecedented precision, thereby overcoming the limitations of earlier estimates, which were primarily based on small sample sets.^30,31,36–38^

It should be emphasized that the term “melt memory” employed in this manuscript, which refers to the memory of a previous crystalline state in the sample as explained in detail above, does not refer to the slow equilibration of entanglement density reported for nascent partially entangled ultrahigh molecular weight polyethylene subjected to annealing.^39–43^

## 2 Experimental Section

### 2.1 Materials

In addition to the previously studied samples reported in our work,^13^ 14 newly synthesized PCL samples with varying molecular weights, covering the low-molecular-weight regime (*M*_w_ ≤ 13 kg/mol) and specifically prepared for this study, were analyzed. Detailed synthesis procedures are provided in the Supporting Information (SI). In our case, using benzyl alcohol (BnOH) as the initiator, the resultant PCL molecules have an α-benzyloxy terminal of a PCL chain (α-benzyloxy ω-hydroxy PCL). The number-average molecular weights (*M*_n_) of the samples were determined by size-exclusion chromatography (SEC) in THF at 35 °C, with data corrected using the Mark–Houwink parameters for PCL (*K* = 1.09 × 10^–3^ dL/g and *a* = 0.6). The *M*_w_, *M*_n_ values, and dispersity indices (*Đ*) for all studied PCL samples are summarized in Table S1, while the corresponding data for the other polar polymer (PEO) are summarized in Table S2. All PEO samples were purchased from Agilent Technologies and marketed as SEC calibration standards. Ultrahigh molecular weight polyethylene (UHMWPE), commercially available as GUR 4120, was obtained from Celanese.

All materials are denoted with superscripts indicating the average molecular weight (*M*_w_) in kg/mol; e.g., PCL^7.15^ denotes a PCL sample with *M*_w_ = 7.15 kg/mol.

### 2.2 Methods

*Differential Scanning Calorimetry (DSC)* analyses were conducted using a PerkinElmer DSC 8500 instrument for PCL. Measurements were performed under an ultrahigh-purity nitrogen atmosphere, flowing at 20 mL/min. The instrument was calibrated with indium and tin standards. Approximately 7 mg of each sample was sealed in DSC pans for analysis. The PEO data were obtained using a TA Instruments DSC 250 under a nitrogen flow of 50 mL/min.^13^ About 1–1.5 mg were used, and the material was dried before measurements.

#### 2.2.1 Standard DSC Scans

To eliminate the thermal history, samples were first heated to *T*_m_ + 30 °C (first heating scan) and held at that temperature for 3 min. They were then cooled to −40 °C at a rate of 20 °C/min while recording the cooling scans. Subsequently, the samples were reheated to *T*_m_ + 30 °C at the same rate (second heating scan) to capture the final heating scans. From these measurements, the crystallization temperature (*T*_c_) and melting temperature (*T*_m_), along with their associated enthalpies, were determined. The degree of crystallinity (*X*_c_) was calculated using eq 1.

$$x_{C}=\frac{\Delta H_{m}}{\Delta H_{m}^{0}}\times 100\%$$ 1

where Δ*H*_m_ is the measured melting enthalpy, and Δ*H*_m_^0^ is the melting enthalpy of fully crystalline polymer, 139.5 J/g for PCL^44^ and 214 J/g for PEO.^45^

#### 2.2.2 Self-Nucleation (SN) Studies

The melt memory was investigated by DSC self-nucleation experiments, following the original protocol designed by Fillon et al.,^5^ later reviewed by Müller and co-workers.^1,2^ According to the SN protocol schematically described in Figure 1, the sample is first heated to a temperature high enough to erase its thermal history and melt memory, holding it for 3 min (step a), thus achieving an isotropic melt state characterized by relaxed chains with random-coiled conformations (i.e., in *Domain I*). Then, the sample is cooled at 20 °C/min (step b), allowing it to crystallize to achieve a “standard” semicrystalline state, characterized by a standard crystallization peak temperature (*T*_c_). After 1 min at the lowest temperature, the sample is heated at 20 °C/min (step c) to a temperature denoted self-nucleation temperature (*T*_s_) and held there for 5 min (step d). The sample was then cooled at 20 °C/min (step e) to promote crystallization, and it was finally heated at 20 °C/min (step f) to monitor melting. To determine the self-nucleation *Domains,* the cooling scan from *T*_s_ (step e) and the subsequent heating (step f) are analyzed.

**Figure 1:**
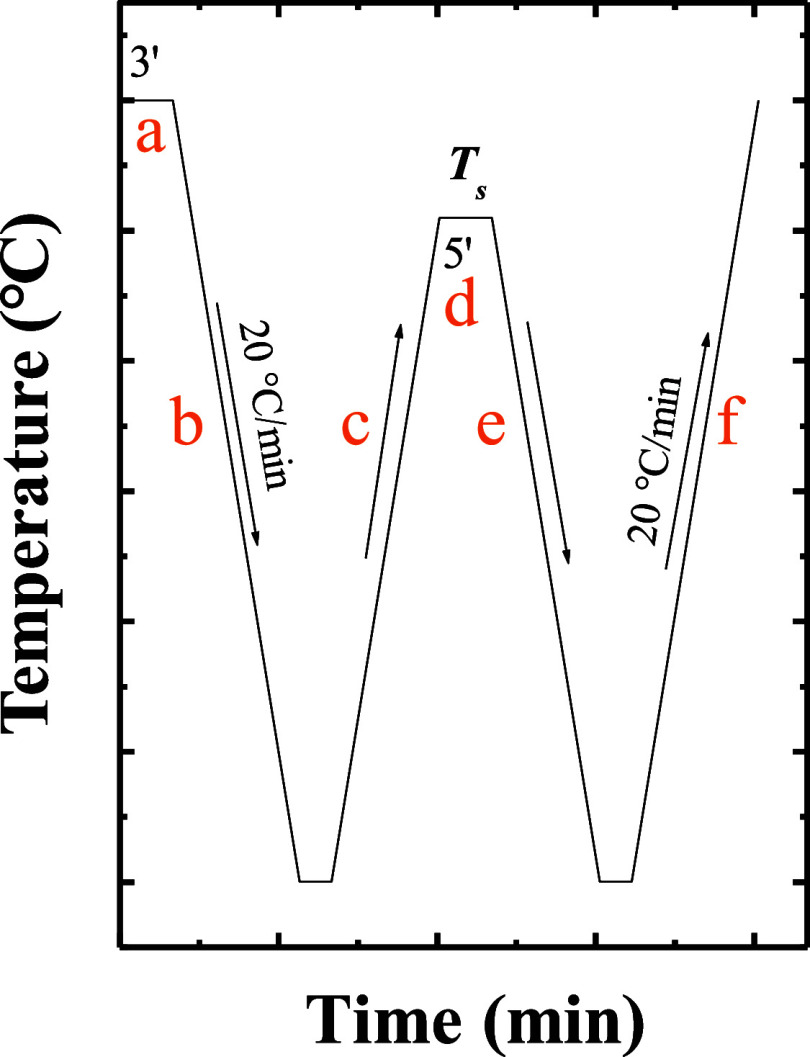
Self-nucleation thermal protocol indicating the steps involved and the cooling/heating rate employed.

#### 2.2.3 Small-Angle X-ray Scattering (SAXS)

Selected samples of PCL were analyzed at room temperature using SAXS at beamline BL11-NCD of the ALBA Synchrotron Radiation Facility (Barcelona, Spain). The X-ray source operated at 12.4 keV (λ = 1.0 Å). Data were collected using a Pilatus 1 M detector (Dectris), which features an active area of 168.7 × 179.4 mm^2^, comprising 981 × 1043 pixels, and a pixel size of 172 × 172 μm^2^. The sample-to-detector distance was set at 6463 mm. Scattering intensity was plotted as a function of the scattering vector, *q* = 4π sin­(θ)*/*λ, calibrated using silver behenate. Samples, placed in standard DSC pans, were mounted in a Linkam THMS-600 hot stage equipped with a liquid nitrogen cooling system. The thermal protocol matched that used in DSC measurements: the material was first heated and then cooled to room temperature at 20 °C/min. The long period (*d*_1_) was obtained from the *q* value corresponding to the maximum peak in the intensity. With the crystallinity value obtained from DSC, it was possible to determine the lamellar thickness (*l*_c_), and amorphous layer thickness (*l*_a_) for each sample.

PEO samples were previously measured at the European Synchrotron Facility (ESRF, Grenoble, France) at the BM26 beamline. The samples were prepared following the thermal procedure employed in the DSC. The long period was calculated from the Lorentz corrected pattern. For more details check reference.^16^

#### 2.2.4 Rheological Measurements

Rheological measurements were performed using an MCR-702 rheometer (Anton Paar, Germany) equipped with a CTD-450 hybrid thermal control unit (combining gas convection and Peltier elements) and operated under a nitrogen atmosphere to minimize oxidation effects. For PEO samples with molecular weight higher than 69.2 kg/mol and PCL samples with molecular weight higher than 62.6 kg/mol, small-amplitude oscillatory shear (SAOS) tests were carried out at temperatures ranging from 70 to 100 °C using an 8 mm parallel-plate geometry. Temperatures above 100 °C were not explored to avoid thermal degradation.

The SAOS data were horizontally and vertically shifted to obtain master curves of the viscoelastic moduli as functions of frequency, according to the time–temperature superposition (TTS) principle (see Section 3.1 for further details). The master curves of PEO^965^, PEO^809^, and PEO^435^ did not exhibit terminal behavior of the viscoelastic moduli within the investigated temperature range. Therefore, creep tests were performed to access that region of the viscoelastic spectrum.

The creep compliance *J*(*t*) was converted into dynamic moduli using the NLREG software based on a nonlinear regularization algorithm.^46^ To ensure that each creep experiment was conducted within the linear regime, several tests were performed at different applied stresses, and the overlap of the resulting creep compliance was verified.

The zero-shear viscosity of the above samples was determined as the limiting value of the complex viscosity as the frequency approached zero. For PEO and PCL samples with lower molecular weights, the zero-shear viscosity was determined from flow curves obtained with 15- or 25 mm parallel-plate geometries. Steady-state viscosity versus shear rate data were collected over temperatures ranging from 60 to 90 °C. The zero-shear viscosity data were fitted in a zero-shear viscosity (η_0_) versus molecular weight curve to obtain the critical molecular weight (*M*_c_) for PEO and PCL, as a further method to confirm the *M*_e_ values obtained from the SAOS tests.

## 3 Results and Discussion

### 3.1 Determination of the Entanglement Molecular Weight, *M*_e_

The entanglement molecular weight (*M*_e_) for both PCL and PEO was determined using two complementary methods: (*i*) plateau modulus analysis and (*ii*) zero-shear viscosity fitting. Measurement temperatures were selected well above *T*_m_, but below 100 °C, to ensure complete melting while minimizing the risk of thermal degradation. As mentioned in Section 2.2.3, SAOS tests were performed on high–molecular weight PEO and PCL samples at different temperatures, and the data were horizontally and vertically shifted to construct master curves. The shift factors are reported in the SI (Figures S5 and S6). Figure 2 presents the master curves in terms of complex viscosity for (a) PEO samples at a reference temperature of 100 °C and (b) PCL samples at a reference temperature of 70 °C. Figure 3 shows the corresponding data in terms of dynamic moduli. The continuous lines in Figures 2a and[Fig fig3]a represent the conversion of the creep compliance into oscillatory data.

**Figure 2:**
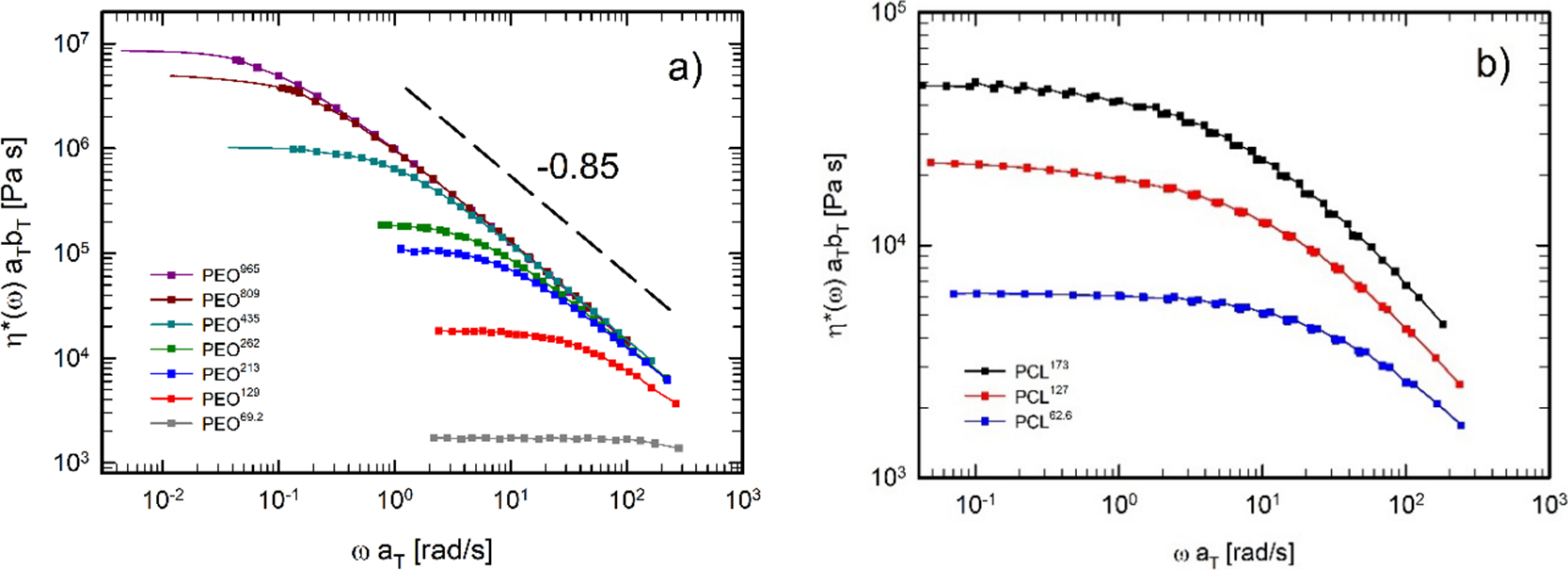
Complex viscosity as a function of frequency for (a) PEO at 100 °C and for (b) PCL at 70 °C. Solid lines represent data obtained from the conversion of the creep compliance into complex viscosity. For PEO, the observed thinning exponent is −0.85, in agreement with the literature.^47,48^

**Figure 3:**
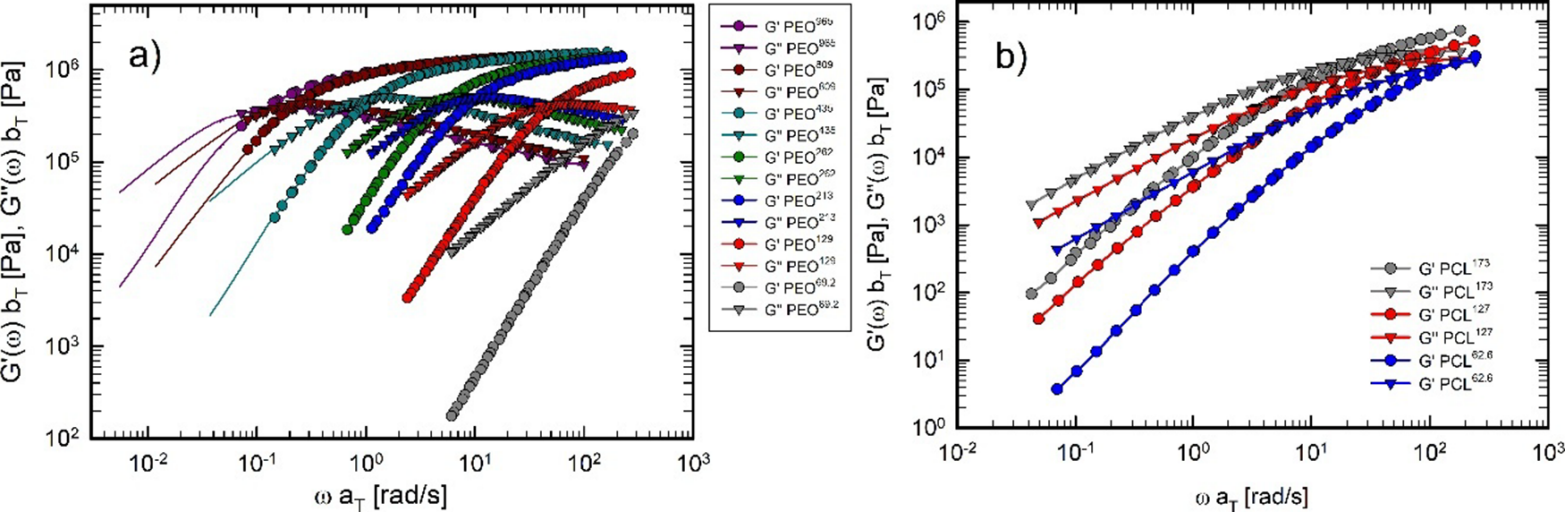
Linear viscoelastic (LVE) master curves of (a) PEO (reference temperature: 100 °C) and (b) PCL (reference temperature: 70 °C). Solid lines represent data obtained from the conversion of the creep compliance into viscoelastic moduli.

The rheological analysis of PEO at 100 °C and PCL at 70 °C reveals clear signatures of entanglement dynamics, as shown by the LVE master curves. Both systems display the expected crossover from terminal to plateau-like behavior, consistent with the formation of an entangled network. The entanglement molecular weight *M*_e_ can be calculated through the value of the plateau modulus *G*_N_^0^ obtained from the master curves in Figure 3, based on the following relationship:^49^

$$M_{e}=\frac{4}{5}\frac{\rho RT}{G_{N}^{0}}$$ 2

where ρ is the polymer density, *R* the gas constant, and *T* the temperature. The value of *G*_N_^0^ is conventionally obtained as the value of the elastic modulus corresponding to the minimum of *G″*.^50^ For this work, however, it was not possible to access the minimum of *G″* (see Figure 3), as crystallization (for both PEO and PCL) did not allow the collection of SAOS data at low temperatures, which correspond to the higher-frequency region of the master curves. For this reason, the estimation of *G*_N_^0^ was carried out using the integral method, based on the following relationship:^50^

$$G_{N}^{0}=\frac{4}{\pi }\times \int_{-\infty }^{\omega_{\max}}G^{\prime \prime }(\omega )d(\ln(\omega ))$$ 3

where ω_max_ is the frequency corresponding to the maximum of *G″*. Equation 3 was used to evaluate the *G*_N_^0^ for the PEO samples PEO^965^, PEO^809^, PEO^435^, PEO^262^, PEO^213^, and PEO^129^ and the PCL samples PCL^173^, PCL^127^, and PCL^62.6^. The calculation was performed through a homemade MATLAB Routine. The average value of *G*_N_^0^ was (1.10 ± 0.05) × 10^6^ Pa for PEO and (9.80 ± 0.20) × 10^5^ Pa for PCL. Such values were inserted in eq 2, to obtain a value of *M*_e_ equal to 2700 ± 120 g/mol for PEO and 2500 ± 50 g/mol for PCL (see Table 1). Assuming that *M*_c_ ≈ 2 *M*_e_, the plateau modulus analysis yielded for PEO *M*_c_ ∼ 5400 g/mol and for PCL *M*_c_ ∼ 5000 g/mol.

**Table 1:** Literature Review of the Values of *M*_e_ Reported for PCL and PEO

material	number of samples used	method	*M*_e_ [g/mol]	*M*_c_ [g/mol]	references
PCL	6	plateau modulus	∼3000	∼6000	^38^
	6		∼3900	∼7800	^37^
	12		∼2500	∼5000	^30^
	3		∼2500	∼5000	this work
	16	zero-shear viscosity fit	2670	5340	this work
PEO	6	H Hahn echo NMR	∼3000	∼6000	^36^
	6	plateau modulus	∼1700	∼3400	^31^
	6		∼2700	∼5400	this work
	18	zero-shear viscosity fit	2785	5570	this work

The zero-shear viscosities η_0_ of (a) PEO and (b) PCL samples for several molecular weights *M*_w_ are reported in Figure 4. The transition from unentangled (oligomeric) to entangled regime is clearly captured by the change in slope of the zero-shear viscosity–molecular weight relationship.

**Figure 4:**
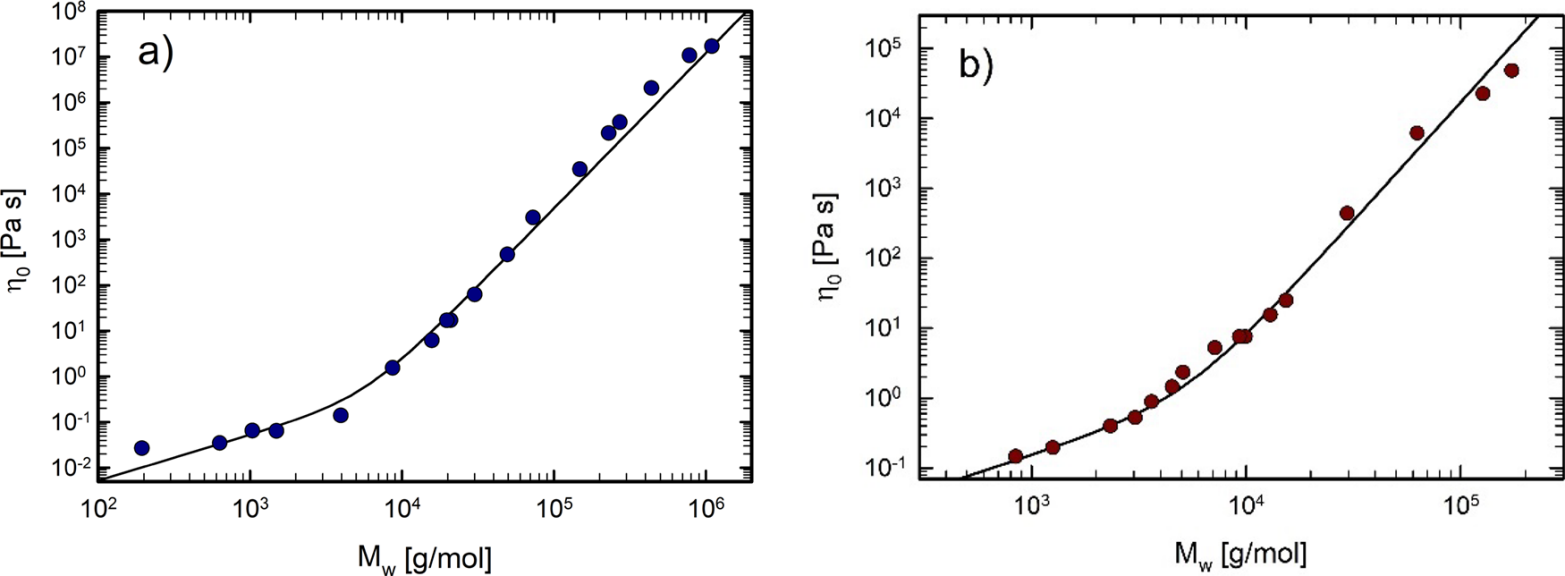
Determination of the critical molecular weight (*M*_c_) from zero-shear viscosity fitting: (a) PEO, yielding *M*_c_ ≈ 5570 g/mol at *T* = *T*_g_ + 125 °C; (b) PCL, yielding *M*_c_ ≈ 5340 g/mol at *T* = *T*_g_ + 130 °C.

The critical molecular weight (*M*_c_) can be obtained by fitting the zero-shear viscosity η_0_ with the relationship proposed by Colby et al.^51^ for linear polymers:

$$\eta_{0}=\alpha^{*}{M_{w}}^{*}(1+(M_{w}/M_{c}{)}^{2.4})$$ 4

where α is an arbitrary constant and *M*_c_ is obtained as a fitting parameter. The viscosity of unentangled chains is proportional to their molecular weight *M*_w_, whereas, for well entangled chains (for *M*_w_ ≫ *M*_c_), the zero-shear viscosity is proportional to *M*_w_^3.4^. It must be mentioned that the coefficient α is influenced by the chain monomeric friction factor, whose value depends on the distance (in terms of temperature) from the polymer glass transition temperature *T*_g_. For this reason, the data reported in Figure 4 have been shifted by the same distance from the glass transition temperature, specifically *T*_g_ + 125 °C for PEO and *T*_g_ + 130 °C for PCL. The glass transition temperatures for PEO were reported by Bailey and Koleske,^52^ while the PCL samples used in this work showed a *T*_g_ consistently near −60 °C by DSC. The zero-shear viscosity fit yielded *M*_c_ equal to 5570 g/mol for PEO and 5340 g/mol for PCL. The corresponding *M*_e_ is 2785 g/mol for PEO and 2670 g/mol for PCL (reported in Table 1).

The determination of *M*_e_ and *M*_c_ for PCL and PEO shows good consistency with previously reported values, while providing improved statistics through larger sample sets and complementary methods (Table 1).

For PCL, literature values of *M*_e_ range from ∼2500 to ∼3900 g/mol and *M*_c_ from ∼5000 to ∼7800 g/mol, primarily obtained via plateau modulus measurements based on eq 2. In this work, both plateau modulus estimation based on eq 2 and zero-shear viscosity fitting based on eq 4 were applied to three and 16 samples, respectively. The range of values obtained is *M*_e_ ≈ 2500–2670 g/mol and *M*_c_ ≈ 5000–5340 g/mol, consistent with prior studies,^30,37,38^ but providing greater coverage of the low-*M*_w_ regime.

For PEO, literature *M*_e_ values span ∼1700–3000 g/mol and *M*_c_ values span ∼3400–6000 g/mol, measured via plateau modulus and Hahn-echo NMR.^31,36^ In this study, the calculation from the plateau modulus for 6 monodisperse samples and the zero-shear viscosity fitting of 18 monodisperse samples yielded *M*_e_ ≈ 2700–2785 g/mol and *M*_c_ ≈ 5400–5570 g/mol, again confirming literature trends while offering a more precise and statistically robust determination.

### 3.2 Calorimetric Properties

The thermal properties of all PCL and PEO samples were analyzed under nonisothermal conditions using differential scanning calorimetry (DSC). The thermal transitions and associated *X*_c_ values obtained from these tests are summarized in Tables S3 and S4. As shown in Table S3, most PCL samples display two distinct melting peaks (*T*_m1_ and *T*_m2_). This double-melting behavior, especially evident in low *M*_n_ PCLs, is typically due to partial melting of less stable, thinner crystals, followed by recrystallization and melting of more stable lamellae during heating.^14,53^ Additionally, consistent with our previous findings and literature reports,^13^ the melting (*T*_m_) and crystallization (*T*_c_) temperatures of PCL, shown in Figure 5a, increase with molar mass, reaching a saturation point at *M*_w_ above 7 kg/mol. This study highlights the trend more clearly due to the larger number of data points in the low *M*_w_ range. For PEO, a similar pattern emerges, with *T*_m_ and *T*_c_ rising with molar mass until around 10 kg/mol, as shown in Figure 5c, and also consistent with our previous findings.^16^ For samples with molar masses below 10 kg/mol, *T*_m_ and *T*_c_ values decrease from the saturation value as the molar mass is reduced due to the limited chain lengths and lamellar thicknesses, and the contribution of chain end effects.

**Figure 5:**
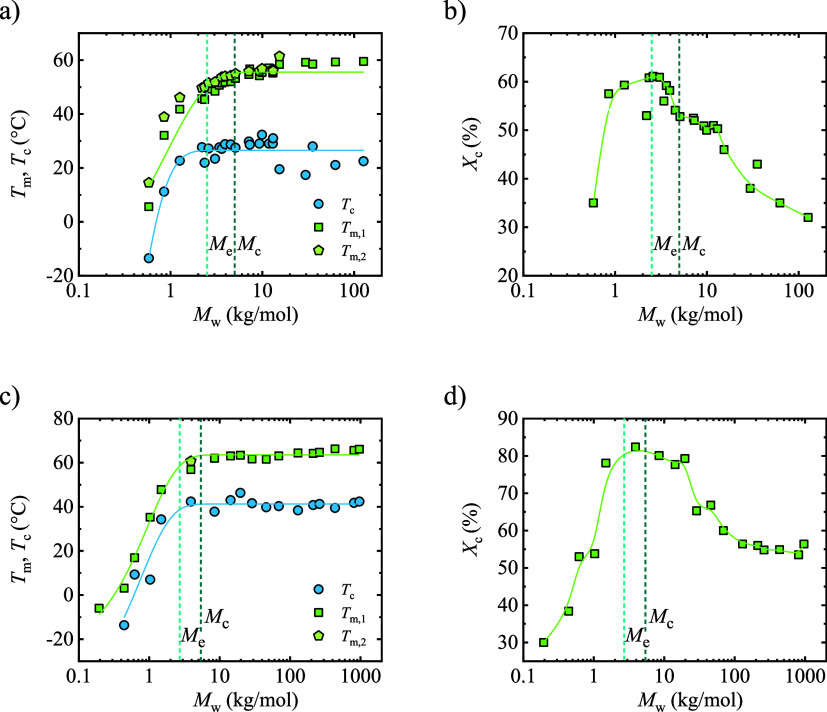
(a) Melting and crystallization temperature as a function of molar mass for PCL, (b) crystallinity degree of PCL, (c) melting and crystallization temperature as a function of molar mass for PEO, and (d) crystallinity degree of PEO.

The degree of crystallinity exhibits a bell-shaped curve when considering molar mass for both polymers, as shown in Figure 5b,d. At very low molar masses, crystallinity is limited by nucleation rather than by diffusion. Short chains possess high mobility; thus, the attachment–detachment balance at the crystal growth front disfavors stable crystal formation, leading to low *X*_c_. As molar mass increases, the attachment probability increases and crystallinity reaches a maximum at approximately 2.4 kg/mol for PCL^13^ and 3.9 kg/mol for PEO. Beyond this maximum, the progressive development and percolation of entanglements restrict chain diffusion, reducing the number of chains able to reorganize into crystalline lamellae during crystallization, and *X*_c_ decreases despite similar nonisothermal crystallization conditions. This evolution of crystallinity is not directly correlated with the crystallization temperature, since *T*_c_ under nonisothermal conditions depends on multiple competing factors (nucleation density, supercooling, and molecular weight) and therefore does not directly reflect the extent of crystallization. The near-constant *T*_c_ observed at high molar masses indicates that comparable supercooling and lamellar thickness are achieved beyond the *M*_c_ limit, even though chain diffusion increasingly limits the number of crystals formed.^13^

The maximum *X*_c_ is approximately 60% for PCL, while PEO can achieve values above 80%. This aligns with the fact that PEO exhibits crystalline interchain diffusion, enabling high degrees of crystallinity as a crystal-mobile polymer. Conversely, PCL does not show notable crystalline interchain diffusion, being crystal-fixed, and thus, exhibits lower crystallinity values.^54,55^

### 3.3 SAXS Measurements

SAXS provides quantitative insight into the lamellar stack architecture of the newly synthesized PCL series. Two complementary length scales were extracted employing the procedure detailed in the SI: the lamellar (crystalline) thickness *l*_c_ and the amorphous layer thickness *l*_a_, and were plotted against *M*_w_ (Figure S2). Above the chain folding threshold for PCL of *M*_w_ ≈ 1.26 kg/mol *l*_c_ remained essentially invariant at ≈6 nm (indicating that the crystalline lamellae reach a saturated thickness once chain folding is established). At values lower than this molecular weight, the samples crystallized in extended chain crystals, and the lamellar thickness increases with *M*_w_, as expected (see Figure S2). On the other hand, *l*_a_ was ≈4 nm for the lowest *M*_w_ samples and increased progressively with *M*_w_, reaching ≈10 nm for the largest chains. This increase in amorphous layer thickness reflects the accommodation of excess chain length and entanglements within the interlamellar amorphous regions rather than changes in lamellar thickness. These trends are consistent with literature reports by Thurn-Albrecht and co-workers^54,55^ indicating PCL behaves as a crystal-fixed polymer, in which increases in molecular weight beyond the folding threshold are primarily accommodated by thickening of the amorphous layers rather than by lamellar thickening.

SAXS data for PCL have been used to estimate the number of folds per chain (*L*/*l*_c_, see Table S5 and eq S3; where *L* is the extended chain length in the trans–trans conformation within the crystal and *l*_c_ is the lamellar thickness). For folded PCL crystals, the crystalline lamellar thickness remains constant at 6 nm, so with this value, *L*/*l*_c_ is calculated as a function of molecular weight. The results show two thresholds: First, a folding threshold occurs near *M*_w_ ≈ 1.26 kg/mol, where *L*/*l*_c_ ≈ 1.1; below this, chains are too short to form a full fold, and the material only forms extended-chain crystals (ECC). Second, as *M*_w_ increases above approximately 1 kg/mol, the number of folds per chain increases steadily, and *l*_a_ grows in parallel. Therefore, the transition from ECC to FCC (folded-chain crystal) is at around 1.0 kg/mol in our PCL series, with the FCC morphology gradually developing more interlamellar amorphous volume as chain length increases.

In the case of PEO, the long period increases with molar mass, as some of us have reported.^16^ For low molar mass samples, the amorphous layer stays constant, while the crystalline lamellar thickness increases. Above 10 kg/mol, the crystalline lamellar thickness stays steady at around 15 nm, but the amorphous layers grow significantly from 5 to 25 nm. For very high molar mass samples, above *M*_n_ 100 kg/mol, the crystalline lamellar thickness increases further from 15 to 25 nm. By comparing the experimentally measured long period with the calculated extended chain length, where PEO chains in the crystal form a 7/2 helical structure, it is possible to determine whether extended or folded chain crystals are formed. The results indicated that extended chain crystals were obtained for samples with a molecular weight of 2 kg/mol or below, as the long period is similar to the value corresponding to the theoretical extended chain length (more details can be found in ref.^16^). On the contrary, folded chain crystals were formed above 2 kg/mol. PEO is a crystal mobile material, this means that intracrystalline chain diffusion occurs within the crystalline phase.^54,55^ This mobility promotes higher crystalline perfection and generally higher crystallinity than in crystal-fixed polymers such as PCL. However, this does not imply a fixed amorphous layer thickness; instead, the amorphous layer thickness in PEO increases with molecular weight as excess chain length and entanglements are accommodated in the interlamellar regions.

### 3.4 Self-Nucleation Studies to Determine Melt Memory in PCL and PEO Samples

The melt memory of the samples was investigated by performing self-nucleation (SN) experiments in the DSC. The analysis of the cooling scan from *T*_s_ (step e, Figure 1) and the subsequent heating scan (step f, Figure 1) allows the determination of the Self-Nucleation *Domains* of the sample, which are shown in Figure 6. The polymer displays the three self-nucleation *Domains* described above, denoted by color code (red for *DI*, blue for *DII,* and green for *DIII*).

**Figure 6:**
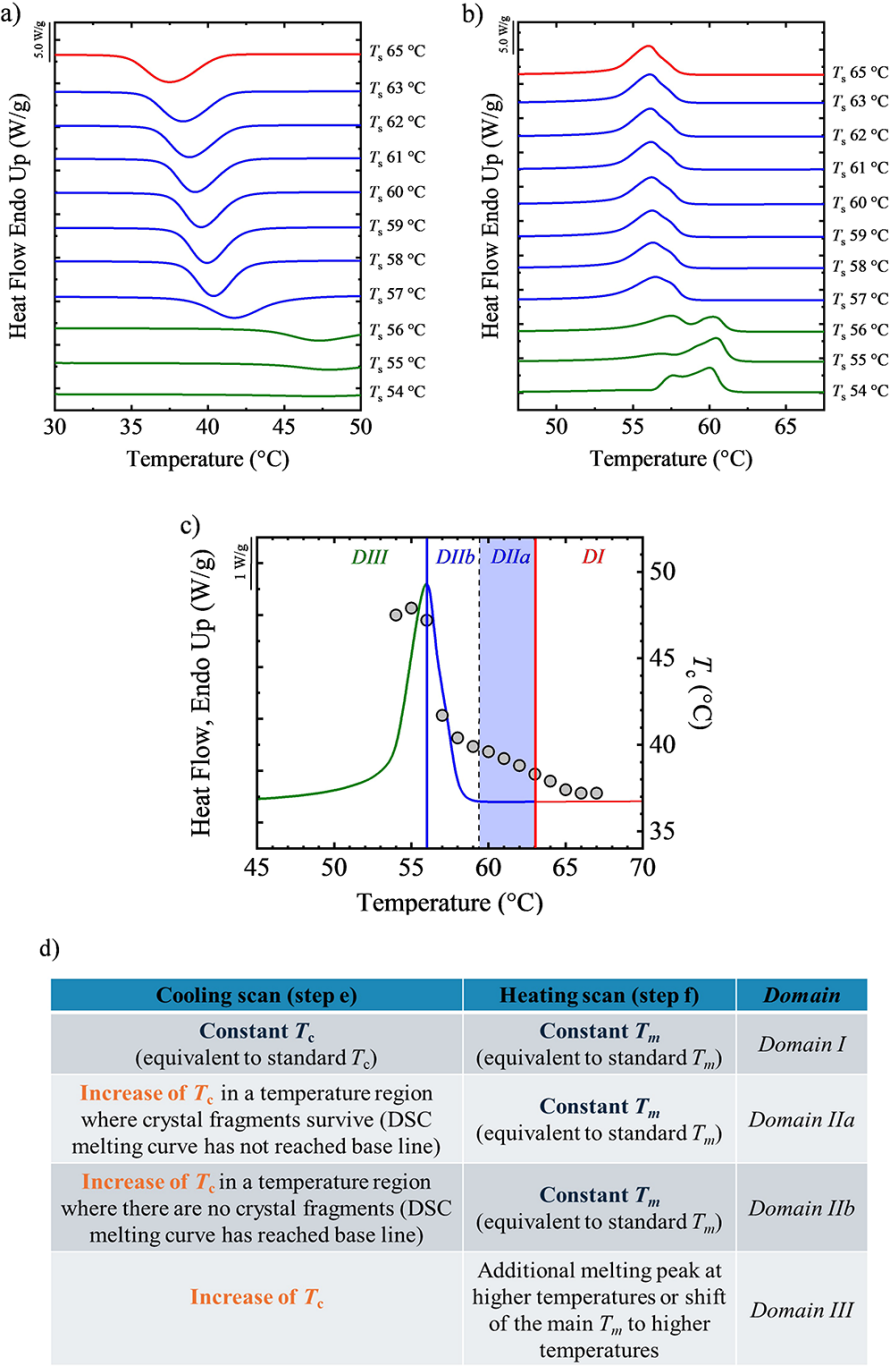
DSC (a) cooling and (b) heating scans after SN at the indicated temperature for PCL^7.15^, (c) SN summary results, the standard heating scan is displayed together with the crystallization temperature obtained at each *T*_s_, and (d) the criteria used to identify the different melt memory *Domains*.

When *T*_s_ is 65 °C or higher, during cooling (Figure 6a, red curve), the material crystallizes at the standard crystallization temperature. This means that the *T*_c_ value obtained during cooling is independent of *T*_s_. Thus, at *T*_s_ = 65 °C or above, an isotropic melt is reached (Figure 6b, red curve), and the sample is in ***Domain I*** or in the ***Isotropic Melting Domain*(*DI*)**.

When *T*_s_ is 63 °C or lower, Figure 6a (blue curves) shows that, during cooling, the material crystallizes at temperatures above the standard value, which increases as *T*_s_ decreases from 63 to 57 °C. At these *T*_s_, some self-nuclei remain in the melt, increasing nucleation density and raising the crystallization temperature during cooling. In this case, the sample is in the ***Self-nucleation Domain*** (*Domain II*, ***DII***). During subsequent heating scans (blue curves in Figure 6b), no large differences in melting behavior are observed between samples in DII and those in DI, indicating that the samples did not anneal during the time spent at *T*_*s*_.

Finally, when the material is heated to *T*_s_ equal or lower than 56 °C (Figure 6b green curves), an increase in the crystallization temperature is observed relative to the standard value. It should be noted that at 56 °C, the sample partially melts, leaving unmolten crystals that can act as self-seeds. More importantly, during subsequent heating (Figure 6b, green curves), an additional melting peak appears at higher temperatures than the standard melting temperature. This additional melting peak corresponds to the unmolten crystals that were annealed. During the time spent at *T*_s_ (step d, Figure 1), unmolten crystals are left intact and undergo annealing with thickening of the crystalline lamellae. Once the material is cooled and subsequently heated, the melting of the crystals formed during cooling from *T*_s_ and thecrystals that were annealed is observed. This is the origin of the bimodal melting endotherms in Figure 6b (green curves). Since annealed crystals have thicker crystalline lamellae, they melt at higher temperatures. In this temperature region, the sample is in the ***Self-Nucleation and Annealing Domain*** or ***Domain III* (*DIII*)*.***

From self-nucleation experiments like those shown in Figure 6, the SN *Domains* for all samples were identified and displayed in Figure 7. The PCL^7.15^ sample displays the three *Domains* mentioned, as illustrated in Figure 6c. Figure 6c presents the standard melting endotherm of PCL^7.15^ drawn in colors that correspond to the *Domains*, as indicated above and in Figure 6a,b, with vertical lines indicating the divisions between *Domains*. Superimposed on this calorimetric plot, we have plotted the experimentally determined peak crystallization temperatures (*T*_c_ values) on the right-hand *y-axis* of Figure 6, using the temperature axis as the *x-axis* and representing, for the plotted data points (gray circles), the self-nucleation temperatures (*T*_s_) applied (data collected from Figure 6a). A summary of the criteria used to identify the melt memory *Domains* is included in Figure 6d.

**Figure 7:**
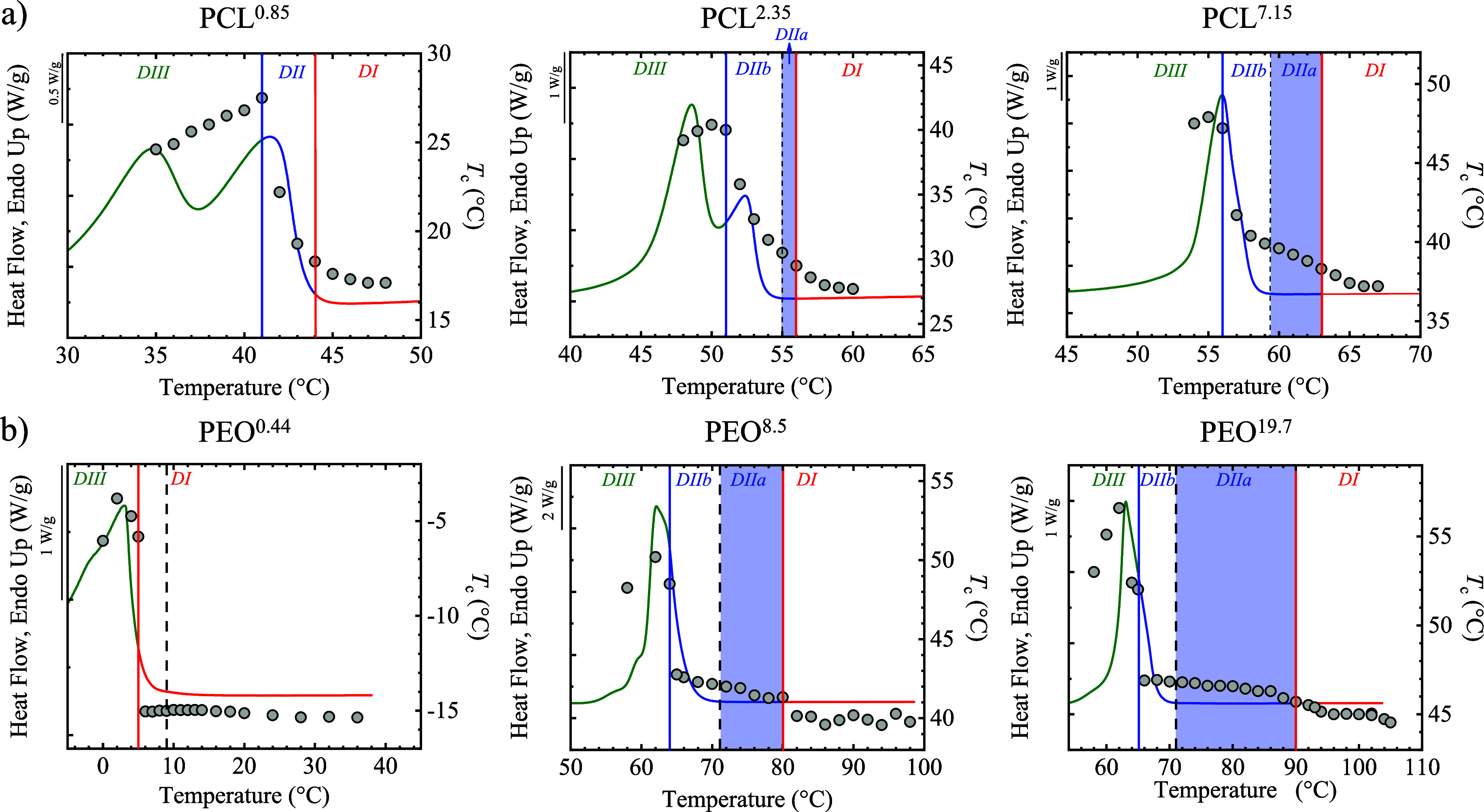
(a) Self-nucleation (SN) *Domains* of PCL: PCL^0.85^ (ECC), PCL^2.35^ (unentangled FCC), and PCL^7.15^ (entangled FCC), superposed on the standard DSC heating scan. The corresponding crystallization temperatures (*T*_c_) are plotted on the right *y*-axis as a function of *T*_s_. (b) SN *Domains* of PEO: PEO^0.44^ (ECC), PEO^8.5^ (entangled FCC), and PEO^19.7^ (entangled FCC), superposed on the standard DSC heating scan. *T*_c_ values are likewise plotted on the right *y*-axis versus *T*_s_.

From plots like those in Figure 6c we can appreciate the division of *Domain II* into two regions, following the analysis by Müller and co-workers.^1,2^ Considering the end of the melting endotherm in Figure 6c, i.e., the temperature at which the DSC heat flow curve reaches the baseline, we can distinguish *Domain IIb* and *Domain IIa* regions. Above *T*_m,end_ (59.5 °C in the example of Figure 6c), the heat flow baseline is reached, which implies that there are no crystals left in the melt that can serve as self-seeds. Therefore, if for *T*_s_ temperatures above *T*_m,end_ an increase in *T*_c_ occurs during cooling (as is the case shown in Figure 6c), such enhanced nucleation can only be due to melt memory. As indicated in Figure 6c, the blue-shaded region of *T*_s_ temperatures corresponds to the ***Melt Memory Domain***, or ***Domain IIa***. On the other hand, when the *T*_*s*_ temperatures employed are below *T*_m,end_, some crystal fragments remain that cannot be annealed; as demonstrated during subsequent heating (step f, Figure 1), and only the standard melting curves are obtained (Figure 6b, blue curves in the temperature region of 59–57 °C). This temperature region is known as ***Domain IIb*** *,* and the crystals too small to anneal are called self-seeds; thus, the sample is in the *Self-Seeding Domain*.

Figure 7 summarizes the SN results, showing how *T*_c_ evolves as a function of *T*_s_ for selected samples of PCL^0.85^, PCL^2.35^, PCL^7.15^, PEO^0.44^, PEO^8.5^, and PEO^19.7^. In some cases, additional data from our previous work was used.^13,16^ Samples with suitable molar mass were selected to illustrate the characteristics of three cases: (a) unentangled extended chain crystals (ECC) for both PEO and PCL, (b) unentangled folded chain crystals (FCC) for PCL, and (c) entangled FCC for both PEO and PCL.

Case (a) is represented in Figure 7 by the low molar mass samples, PCL^0.85^ (*M*_w_ = 0.85 kg/mol) and PEO^0.44^ (*M*_w_ = 0.44 kg/mol), which form extended chain crystals and the chains remain unentangled, as they are below the determined *M*_e_ values. The SN results indicate that in some low *M*_w_ samples (like in PEO^0.44^), no detectable *Domain II* region exists, while in others, no melt memory can be observed (i.e., absence of *DIIa*). Most importantly, across all results (see below and Figure 8), there is a critical chain length below which unentangled samples (which form ECC) do not exhibit melt memory, in both PEO and PCL. SAXS confirmed that these low *M*_w_ PCL samples without melt memory have *L*/*l*_c_ < 1. In the case of PEO, extended chain crystals are also formed, with the chains remaining unentangled, as indicated by the experimental value of *M*_e_ obtained in this work.

**Figure 8:**
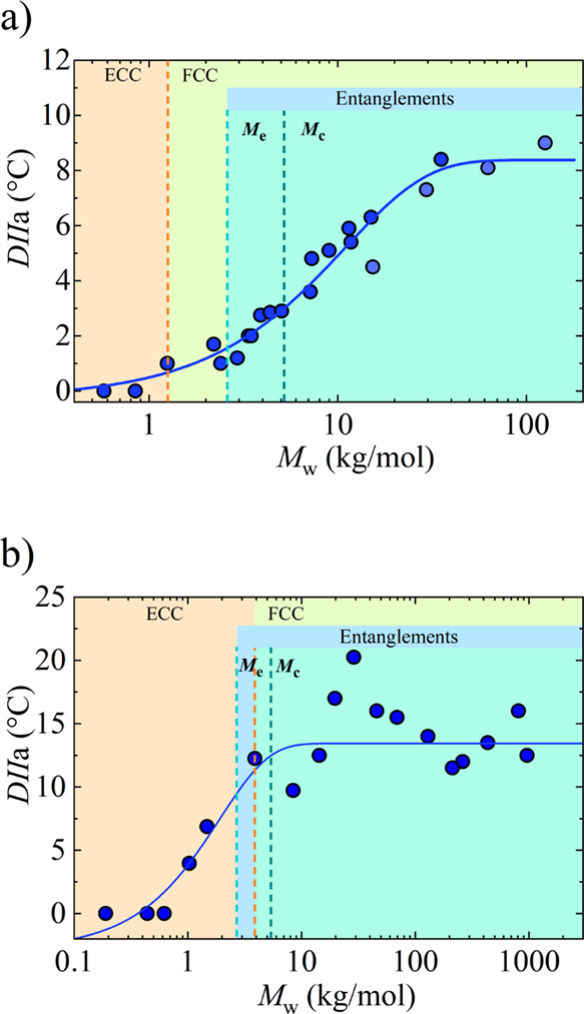
Strength of melt memory as revealed by the width of *Domain IIa* (*DIIa*) as a function of weight-average molecular weight for (a) PCL and (b) PEO.

Case (b) of unentangled folded chain crystals (FCC) for PCL is shown in Figure 7, using PCL^2.35^ as an example, which exhibits a well-defined, narrow *Domain IIa* region. SAXS indicates that these chains form folded lamellae with an *L*/*l*_c_ ratio of 1.8 (see Table S5), close to two folds per chain. Rheological analysis shows that PCL^2.35^ is just below the entanglement melting point *(M*_e_ *)*. The presence of melt memory below *M*_e_ (also observed in the once-folded chain sample PCL^1.26^, see Table S5 and Figure 8 below) suggests that when *M*_w_ exceeds about 1 kg/mol, the number of intermolecular interactions becomes high enough to induce melt memory. In other words, for PCL, the critical chain length for melt memory development below the entanglement molecular weight is around 1 kg/mol. These intermolecular interactions in PCL result from the combined effects of its modest dipolar interactions (C = O···H–C, C = O···C=O)^33^ and the crystallinity developed. The next section will analyze the quantitative calculation of an interaction index that influences melt memory.

Case (c) for the entangled FCC PEO and PCL is also shown in Figure 7. The strongest melt memory appears in entangled FCC samples, such as PCL^7.15^, PEO^8.5,^ and PEO^19.7^. Here, *Domain IIa* broadens significantly, indicating a stronger melt memory, with the critical *T*_s_ needed to erase memory shifting upward compared to unentangled FCC for PCL or unentangled ECC for PEO. SAXS shows that for PCL and PEO, while lamellar thickness stays constant, *l*_a_ increases further. Rheological analysis confirms that these samples are well above *M*_e_, indicating that interchain entanglements topologically constrain their amorphous layers. The combination of multiple chain folds and numerous entanglements slows melt relaxation, enhancing melt memory as the melt becomes topologically more complex.

Figure 8 illustrates the width of the melt memory *Domain IIa* as functions of molecular weight for both PCL and PEO. The experimentally determined *M*_e_ values for PCL and PEO are listed in Table 1 and are shown in Figure 8 as vertical segmented lines. The corresponding critical molecular weights, at which entanglements form a network connecting all chains in the melt (*M*_c_ *≈ 2M*_e_), are approximately 5–6 kg/mol for PCL and 5–5.5 kg/mol for PEO. The number of entanglements (*Z*) and the number of folds (*n*) as a function of molar mass are shown in the SI (Figure S3).

It is clear from Figure 8 that melt memory begins beyond a critical chain length; however, its onset correlates most directly with the appearance of chain folding rather than with the development of a fully entangled melt. In the case of PCL, the first sample that shows melt memory has an *M*_w_ of 1.26 kg/mol (with a *DIIa* width of only 1 °C) and FCC morphology (*n* > 1), indicating that the formation of chain folds is the primary trigger for melt memory below *M*_e_. While for PEO, it corresponds to a sample with *M*_w_ of 1 kg/mol (with a *DIIa* width of 3.5 °C) that still belongs to the ECC regime (*n* ≈ 1), reflecting the additional contribution of strong intermolecular interactions in this polymer.

The melt memory increases with molecular weight in both samples, though it is more gradual in PCL and becomes more pronounced once the *M*_c_ threshold is exceeded. For PEO, the melt-memory width exhibits a pronounced peak around 10–30 kg/mol due to the combined effect of high crystallinity and rising entanglement density, before leveling off at higher molecular weights, whereas in PCL it occurs at 50 kg/mol. Additionally, the maximum width in *Domain IIa* is higher for PEO (about 12.5 °C at the plateau, with some samples ranging from 12.5 to 20 °C) than for PCL (8 °C).

The results shown in Figure 8 indicate that the critical length for the emergence of melt memory is slightly shorter for PEO than for PCL. PEO can exhibit substantial melt memory when forming extended chain crystals with unentangled chains (up to 7 °C), while PCL only begins to show melt memory (1 °C) at the start of chain folding. As the number of entanglements per chain (*Z*) increases (see Figure S3), melt memory also increases, as the melt’s complexity develops further and the temperature required to reach an isotropic state increases. However, in the fully entangled state for both types of polymers, the melt memory of PEO remains higher than that of PCL (as indicated by the saturation value of *DIIa* width).

The general trends discussed above (Figure 8) suggest that PEO exhibits a stronger melt memory than PCL. This difference can be explained by the fact that PEO can form more robust intermolecular interactions in the crystalline state than PCL. The following section proposes a simple calculation to establish an intersegmental/intermolecular interaction index between chain segments.

### 3.5 Calculation of an Interaction Index

To rationalize how intermolecular interactions and melt rheology control melt memory, we introduce a dimensionless interaction index defined as

$$\mathrm{Interaction}\;\mathrm{index}=\frac{(\delta_{p}+\delta_{h})\cdot X_{c}}{G^{0.5}}$$ 5

where δ_p_ and δ_h_ are the Hansen polar and hydrogen-bonding solubility parameters (MPa^1/2^), *X*_c_ is the experimental degree of crystallinity, and *G* is the shear modulus (MPa). As for what concerns the part of the index which takes into account directly intermolecular interactions, we did not consider the dispersion component (δ_d_), as δ_d_ is essentially constant across PCL, PEO, and PE due to their similar methylene-dominated backbones, and therefore does not contribute significantly to differences in intermolecular interactions relevant to melt memory. This formulation is proportional to the effective density of intermolecular interactions within the crystalline regions (since it is proportional to the cohesive energy density) and, thus, should also correlate with their persistence in the melt.

We have employed the following values obtained by Abbott:^56^

$$\mathrm{PCL}:\delta_{p}(5\;{\mathrm{MPa}}^{1/2})+\delta_{h}(8.4\;{\mathrm{MPa}}^{1/2})=13.4\;{\mathrm{MPa}}^{1/2}$$

$$\mathrm{PEO}:\delta_{p}(10\;{\mathrm{MPa}}^{1/2})+\delta_{h}(5\;{\mathrm{MPa}}^{1/2})=15\;{\mathrm{MPa}}^{1/2}$$

$$\mathrm{PE}:\delta_{p}(0.8\;{\mathrm{MPa}}^{1/2})+\delta_{h}(2.8\;{\mathrm{MPa}}^{1/2})=3.6\;{\mathrm{MPa}}^{1/2}$$

Even without considering the degree of crystallinity of the samples, the above values show that the cohesive energy density of PEO (i.e., the square root of δ) is higher than that of PCL; therefore, for the same number of crystalline regions, the intermolecular interactions will be stronger for PEO.

The interaction index as a function of molar mass has been calculated using the above-mentioned Hansen solubility parameter values, the crystallinity degree obtained from DSC (Tables S2 and S4), and the shear modulus as explained below.

The shear modulus *G* of a polymer has an entropic origin and is directly linked to the number of elastically active elements per unit volume:

$$G=\nu k_{B}T=\frac{\rho RT}{M_{s}}$$ 6

where ν is the number density of elastically active strands, *k*_B_ the Boltzmann constant, *T* the temperature, ρ the polymer density, *R* the gas constant, and *M*_s_ is the molecular weight of the elastically active strand.

Depending on the molecular weight of the polymer chains, two regimes can be distinguished. When chains are short enough, their dynamics are described by the Rouse model. In this case, the elastically active element is the *entire chain*, which contributes an entropic energy of the order of *k*_B_ *T* to macroscopic elasticity. Therefore, *M*_s_ corresponds to the molecular weight of the whole chain, and the modulus decreases inversely with increasing chain molecular weight.

When the chain length exceeds the entanglement threshold, the elastically active element becomes the *strand between entanglements*, so *M*_s_ = *M*_e_. Since *M*_e_ is a material constant, the modulus becomes independent of molecular weight and reaches the characteristic plateau value *G*_N_^0^.

The inverse modulus is proportional to 1/ν, which represents the average volume associated with one elastically active strand. A small value of 1/ν corresponds to a high density of constraints, whereas a large 1/ν corresponds to a lower density of constraints. Therefore, if one wants to account for the constraint effect, the interaction parameter should scale with 1/ν, that is, with 1/*G*, because the higher the packing, the greater the likelihood of local interactions in the melt. To make the index dimensionless, we decided to make it proportional to 1/*G* ^0.5^. The values of the shear modulus are reported in Tables S3 and S4.

The results are plotted in Figure 9 for PCL and PEO. The trend with molar mass follows the same behavior displayed by the crystallinity degree (Figure 5), as expected. PCL displays a maximum in the interaction index at around 3 kg/mol, with values close to 7. The only sample with a low index in the low-molar-mass region is PCL^0.58^. Above 3 kg/mol, the index decreases from 7 to 4 due to a decrease in crystallinity.

**Figure 9:**
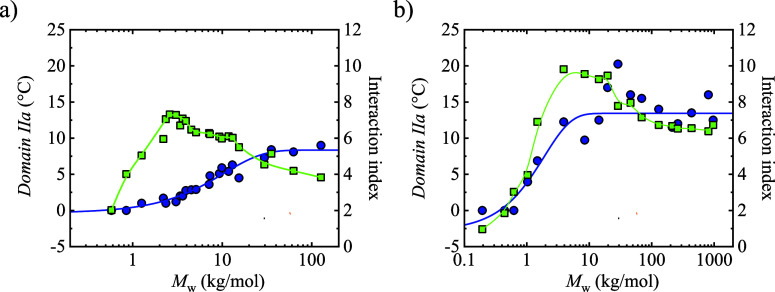
Interaction index (green symbols, right-hand side *y*-axis) and *Domain IIa* (blue symbols, left-hand side *y*-axis) as a function of molar mass for (a) PCL and (b) PEO.

PEO exhibits a similar trend, with a maximum interaction index of 10 for samples around 4 kg/mol, which display the highest degree of crystallinity, and then decreasing to 6.5 as the molar mass increases, and the crystallinity is reduced. The behavior of the interaction index with molar mass is similar for both polymers, but the absolute values remain consistently higher for PEO than for PCL because the ether oxygen groups in PEO provide stronger polar interactions than the ester groups in PCL.

Together, these results highlight a dual control mechanism for melt memory in polar homopolymers: (i) above *M*_e_, the entanglements hinder reaching an isotropic melt at temperatures around the melting peak, aligning with rheology-based interpretations; and (ii) below *M*_e_, the increase in interaction index due to intermolecular interactions and higher crystallinity provides an alternative route for enhancing melt memory, even without entanglements, which makes it more difficult to achieve an isotropic melt.

Comparison of PCL and PEO shows that for PEO, melt memory appears even in extended-chain crystals, whereas for PCL, folded-chain crystals are required to display melt memory. In addition, if high molar mass samples are compared, PEO displays a broader *Domain IIa* than PCL (≈20 °C for PEO vs ≈9 °C for PCL). This can be explained by the higher interaction index of PEO (∼9 at typical crystallinity) compared with that of PCL (∼7). The stronger dipole density in PEO, combined with its high crystallinity, therefore enhances melt memory relative to PCL.

Finally, we compared both PCL and PEO to a purely apolar polymer (PE in this case, with δ_p_ + δ_h_ ≈ 3.6 MPa^1/2^), which has similar chain flexibility but lacks significant polar interactions. Our SN studies on UWMWPE showed only a very narrow *Domain II* (∼1 °C; Figure S4), caused solely by self-seeding, the typical self-nucleation mechanism of apolar, mainly linear polymers. In this context, Balsamo et al.^57^ demonstrated that LDPE exhibits the standard three-*Domain*s self-nucleation behavior, in which *Domain II* arises solely from residual crystalline fragments (i.e., self-seeding), without melt memory. In contrast, the presence of polar groups in polymers like PCL and PEO can greatly enhance melt memory. Comparing PCL and PEO with PE effectively isolates the influence of polarity from that of entanglements, confirming that polar/hydrogen-bonding interactions are essential for melt memory to develop in linear homopolymers. Without these interactions, even a highly entangled polymer like UHMWPE cannot develop melt memory. Conversely, if intermolecular interactions are present, entanglements can serve as stabilizers and enhancers of melt memory, increasing melt relaxation times and adding complexity to the melt state.

## 4 Conclusions

We present a comprehensive framework for understanding the molecular origins of melt memory in polar semicrystalline homopolymers by combining rheology, SAXS, and DSC-based self-nucleation analysis across a well-defined series of PCL and PEO spanning molecular weights from the oligomeric regime to well above the entanglement molecular weight.

SAXS measurements reveal the molar masses at which chains begin to fold within the crystals: 1.26 kg/mol for PCL and 2.0 kg/mol for PEO. The results show that the crystalline lamellar thickness stays constant beyond the chain folding threshold, while the amorphous layer increases with molar mass at intermediate values. Our rheological study, performed with a large number of samples, provided accurate entanglement molecular weight values of *M*_e_, of approximately 2.5–2.7 kg/mol for both polymers. Two rheological techniques were used: viscoelastic measurements and the analysis of zero-shear viscosity versus molar mass, producing similar results.

The study of self-nucleation behavior for the two polar polymers used here shows that melt memory appears in samples without entanglements and grows with molar mass as entanglements develop. Specifically, in PCL, melt memory occurs when chains can fold even in the absence of entanglements. Conversely, for PEO, melt memory is evident even in extended chain crystals and increases as chains begin to fold within the crystal.

Polymers below *M*_e*,*_ i.e., in the absence of entanglements, require strong intermolecular interactions to develop melt memory and thus exhibit higher temperatures than the melting point to reach an isotropic melt. This has been demonstrated by quantifying intermolecular or intersegmental interactions and rheological constraints using an interaction index derived from Hansen solubility parameters, crystallinity, and shear modulus. The crucial role of intermolecular interactions is shown by comparing the results with PE, an apolar polymer that exhibits no melt memory, even at very high molar masses.

Taken together, these findings show that melt memory in polar polyesters and polyethers depends on chain length and intermolecular interactions. Below *M*_e_, intermolecular interactions in the crystalline state are responsible for melt memory in polar homopolymers (which relies on the strength of these interactions and the degree of crystallinity). Above *M*_e_, in addition to intermolecular interactions, entanglements hinder the attainment of an isotropic melt, allowing self-nuclei to survive at high temperatures. By combining these insights, we demonstrate how chain length and intermolecular interactions influence melt memory, offering general principles for tailoring melt memory in semicrystalline polymers through chain length and chemical structure.

## Supplementary Material



## Notes

Synthesis of α-benzyloxy ω-hydroxy poly­(e-caprolactone) (PCL); additional properties calculated from DSC and rheology results; SAXS data; self-nucleation (SN) measurements of ultrahigh-molecular-weight polyethylene; calculation of the interaction index; and rheology (PDF)
