# Supplementary material for: Decoupling the Roles of Chain Length, Entanglements, and Intermolecular Interactions on the Melt Memory of Semicrystalline Polar Homopolymers
Source: Macromolecules. 2026 Mar 7;59(6):3371–83. doi: 10.1021/acs.macromol.5c03323 (PMC13037052; doi:10.1021/acs.macromol.5c03323)
Supplement: Supplementary file 1 [file ma5c03323_si_001.pdf]

SUPPLEMENTARY INFORMATION

Decoupling the roles of chain length, entanglements and intermolecular interactions on the melt memory of semicrystalline polar homopolymers

M. Ali Aboudzadeh[1], Leire Sangroniz[2], Olivier Coulembier[3], Marcello Ferranti[4], Salvatore Costanzo[4], Nino Grizzuti[4], D. Cavallo*[5], Alejandro J. Müller[2,6]**

[1]POLYMAT and Department of Applied Chemistry, Faculty of Chemistry, University of the Basque Country UPV/EHU, Paseo Manuel de Lardizabal 3, 20018, Donostia-San Sebastián, Spain

[2]POLYMAT, Department of Polymers and Advanced Materials: Physics, Chemistry and Technology, Faculty of Chemistry, University of the Basque Country UPV/EHU, Paseo Manuel de Lardizábal, 3, Donostia-San Sebastián 20018, Spain.

[3]Laboratory of Polymeric and Composite Materials, University of Mons, Place du Parc 23, 7000, Mons, Belgium

[4] Department of Chemical, Materials, and Production Engineering (DICMAPI), University of Naples Federico II, P.le Tecchio 80, Naples 80125, Italy

[5]Department of Chemistry and Industrial Chemistry, University of Genoa, Via Dodecaneso 31, 16146, Genoa, Italy.

[6]IKERBASQUE, Basque Foundation for Science, Plaza Euskadi 5, Bilbao 48009, Spain.

**Corresponding authors: salvatore.costanzo@unina.it, dario.cavallo@unige.it, alejandrojesus.muller@ehu.es*

1) Synthesis of α-benzyloxy ω-hydroxy poly(e-caprolactone) (PCL).

Materials

Benzyl alcohol (BnOH) was dried over calcium hydride (CaH_2) for 48 hours and then distilled under reduced pressure. ε-Caprolactone (CL, from Aldrich) was distilled under reduced pressure (80 °C/1 mbar) from CaH_2 and stored over 4 Å molecular sieves. Tin(II) bis(2-ethylhexanoate) ($Sn(Oct)_2$, 95%, from Aldrich) was used as received, without further purification. All compounds were stored in a glove box ($O_2 \leq 6$ ppm, $H_2O \leq 1$ ppm).

General polymerization procedure

In a glove box, a dried vial equipped with a magnetic stir bar was charged with ε-caprolactone (CL), benzyl alcohol (BnOH), and $Sn(Oct)_2$. The initial molar ratio of $[BnOH]_0$ to $[Sn(Oct)_2]_0$ was fixed at 200 while the $[CL]_0/[BnOH]_0$ ratio varied according to the desired degree of polymerization (DP). The sealed vial was then removed from the glove box and heated to 110 °C for a reaction time corresponding to the targeted DP. The corresponding reaction time was 2h for $PCL^{0.58}$ and $PCL^{0.85}$; 3h for $PCL^{1.26}$; 6h for $PCL^{2.35}$ and $PCL^{2.6}$; 7h for $PCL^{3.62}$, $PCL^{3.93}$, $PCL^{4.53}$, $PCL^{5.1}$and $PCL^{7.15}$; 15.5h for $PCL^{9.3}$ and $PCL^{9.92}$; 20h for $PCL^{13.0}$ and $PCL^{13.1}$.

Upon completion, the reaction mixture was cooled to 25 °C, dissolved in THF, and precipitated into cold heptane. The resulting PCL was collected by filtration and dried under reduced pressure at 40 °C until a constant weight was achieved. Table S1 summarizes M_w, M_n, and dispersity ($Đ$) of all PCL samples, including the new ones, while the corresponding data for PEO are shown in Table S2. All PEO samples were purchased from Agilent Technologies.

Table S1. M_w, M_n and $Đ$ values of the studied PCL samples[1]

Sample	***M_w (Kg/mol)***	***M_n (Kg/mol)***	***Đ***	***Sample***	***M_w (Kg/mol)***	***M_n (Kg/mol)***	***Đ***
$PCL^{0.58}$	0.58	0.48	1.2	$PCL^{7.15}$	7.15	5.5	1.3
$PCL^{0.85}$	0.85	0.65	1.3	$PCL^{7.3}$	7.3	5.6	1.3
$PCL^{1.26}$	1.26	0.96	1.3	$PCL^{9.3}$	9.3	7.5	1.2
$PCL^{2.2}$	2.2	2	1.1	$PCL^{9.92}$	9.92	8.2	1.2
$PCL^{2.35}$	2.35	1.6	1.5	$PCL^{11.8}$	11.8	8.4	1.4
$PCL^{2.6}$	2.6	2.2	1.2	$PCL^{13.0}$	13.0	9.4	1.4
$PCL^{3.05}$	3.05	2.1	1.4	$PCL^{13.1}$	13.1	8.8	1.5
$PCL^{3.4}$	3.4	2.4	1.4	*$PCL^{15.4}$*	15.4	9.6	1.6
$PCL^{3.62}$	3.62	2.9	1.2	*$PCL^{29.6}$*	29.6	17.4	1.7
$PCL^{3.93}$	3.93	3.25	1.2	$PCL^{35.4}$	35.4	22.1	1.6
$PCL^{4.53}$	4.53	3.65	1.2	*$PCL^{62.6}$*	62.6	34.8	1.8
$PCL^{5.1}$	5.1	4.2	1.2	*PCL^{127}*	126.9	70.5	1.8

[1] Commercial samples are marked in *italic* letters.

Table S2. M_w, M_n and $Đ$ values of the studied PEO samples[1]

Sample	*M_w (Kg/mol)*	*M_n (Kg/mol)*	*Đ*	*Sample*	*M_w (Kg/mol)*	*M_n (Kg/mol)*	*Đ*
$PEO^{0.194}$	0.194	0.194	1.0	$PEO^{28.8}$	28.8	27.4	1.05
$PEO^{0.44}$	0.44	0.40	1.08	$PEO^{45.9}$	45.9	42.9	1.07
$PEO^{0.62}$	0.62	0.58	1.07	$PEO^{69.2}$	69.2	66.0	1.05
$PEO^{1.03}$	1.03	0.98	1.05	PEO^{129}	129.5	118.8	1.09
$PEO^{1.48}$	1.48	1.44	1.03	PEO^{213}	213.1	195.5	1.09
$PEO^{3.9}$	3.9	3.75	1.04	PEO^{262}	261.7	249.2	1.05
$PEO^{8.46}$	8.46	8.13	1.04	PEO^{435}	434.7	414	1.05
$PEO^{14.3}$	14.3	13.4	1.07	PEO^{809}	809.3	763.5	1.06
$PEO^{19.7}$	19.7	18.4	1.07	PEO^{965}	964.6	869	1.11

2) Additional properties calculated from DSC and rheology results

Table S3. Summary of thermal transition temperatures, enthalpies of crystallization and fusion, and degree of crystallinity (X_c) values obtained from non-isothermal DSC measurements for all PCL samples analysed in this study. The shear modulus and the dimensionless Interaction Index are also listed.

Sample Name	*Cooling scan*		*Heating scan*				*Shear Modulus*	*Interaction index*
	T_c (°C)	ΔH_c (J/g)	T_{m1} (°C)	T_{m2} (°C)	ΔH_m (J/g)	X_c (%)	G (MPa)	$(\delta_p+\delta_h) \times X_c \times G^{-0.5}$
$PCL^{0.58}$	-13.5	49	5.6	14.5	48	35	5.41	2.02
$PCL^{0.85}$	11.2	82.0	32.1	38.9	80.1	57.5	3.69	4.01
$PCL^{1.26}$	22.7	85.1	41.8	46.1	82.6	59.3	2.50	5.04
$PCL^{2.2}$	27.7	74	45.7	49.7	76	53	1.43	5.95
$PCL^{2.35}$	21.9	82.5	45.4	50.0	84.7	60.8	1.33	7.05
$PCL^{2.6}$	27.2	83.2	49.3	51.5	85.1	61.1	1.25	7.31
$PCL^{3.05}$	23.4	82.6	48.5	51.9	84,9	60.9	1.25	7.28
$PCL^{3.4}$	27.6	78	50.7	-	79	56	1.25	6.70
$PCL^{3.62}$	27.1	79.7	51.5	53.7	82.4	59.2	1.25	7.08
$PCL^{3.93}$	28.8	80.9	51.8	54.0	81.1	58.2	1.25	6.96
$PCL^{4.53}$	28.7	79.5	52	54.2	75.3	54.1	1.25	6.47
$PCL^{5.1}$	27.5	77.5	53.2	55	73.6	52.8	1.25	6.32
$PCL^{7.15}$	29.8	76.2	54.7	55.8	73.1	52.5	1.25	6.28
$PCL^{7.3}$	28.6	73	56.7	-	72	52	1.25	6.22
$PCL^{9.3}$	29.0	73.8	54.2	56	70.9	50.9	1.25	6.09
$PCL^{9.92}$	32.3	75.3	55.4	56.8	69.6	50.0	1.25	5.98
$PCL^{11.8}$	29.0	71	57.1	-	71	51	1.25	6.10
$PCL^{13.0}$	29.0	75.1	55.2	56.6	70	50.3	1.25	6.02
$PCL^{13.1}$	31.0	72.3	55.4	56.0	70	50.3	1.25	6.02
$PCL^{15.4}$	19.5	65	58.4	61.4	68	46	1.25	5.50
$PCL^{29.6}$	17.4	54	59.2	-	56	38	1.25	4.54
$PCL^{35.4}$	28.0	60	58.5	-	60	43	1.25	5.14
$PCL^{62.6}$	21.1	49	59.3	-	50	35	1.25	4.19
PCL^{127}	22.5	44	59.5	-	45	32	1.25	3.83

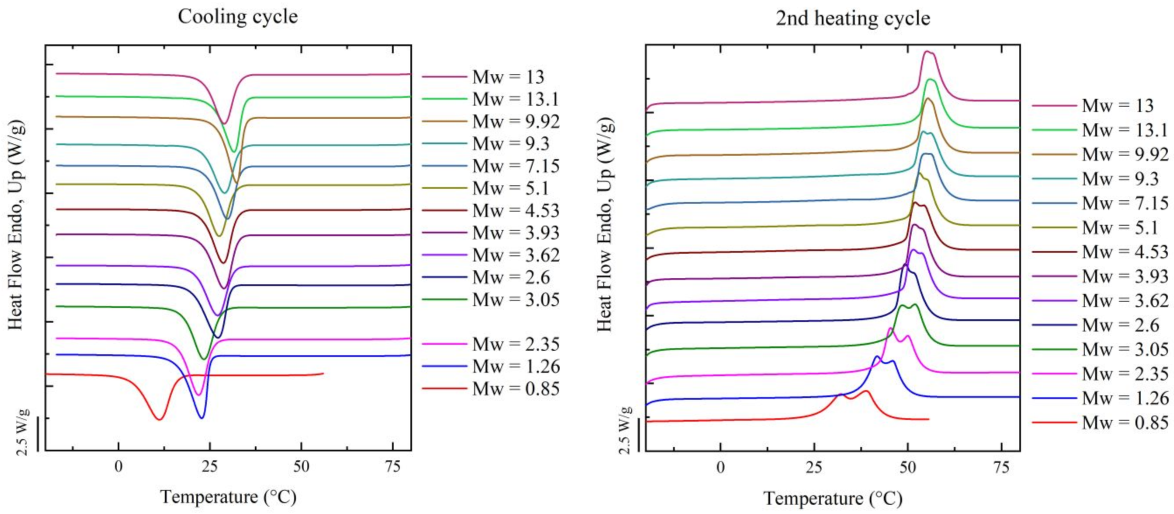

Figure S1. Raw DSC plots for the new PCL samples synthesized in this study.

Table S4. Summary of thermal transition temperatures, enthalpies of crystallization and fusion, and degree of crystallinity (X_c) values obtained from non-isothermal DSC measurements for all PEO samples analysed in this study. The shear modulus and the dimensionless Interaction Index are also listed.

Sample Name	*Cooling scan*		*Heating scan*				*Shear Modulus*	*Interaction index*
	T_c (°C)	ΔH_c (J/g)	T_{m1} (°C)	T_{m2} (°C)	ΔH_m (J/g)	X_c (%)	G (MPa)	$(\delta_p+\delta_h) \times X_c \times G^{-0.5}$
$PEO^{0.19}$			-6.0		64.3	30.0	17.58	0.96
$PEO^{0.44}$	-13.7	83.8	3.1		82.2	38.4	7.75	1.85
$PEO^{0.62}$	9.3	113.4	16.9		113.5	53.0	5.50	3.03
$PEO^{1.03}$	7.0	119.6	35.3		115.2	53.8	3.31	3.96
$PEO^{1.48}$	34.3	163.6	47.8		167.0	78.1	2.30	6.90
$PEO^{3.9}$	42.4	170.1	56.9	60.7	176.3	82.4	1.26	9.82
$PEO^{8.46}$	37.8	164.5	62.0		171.5	80.1	1.26	9.55
$PEO^{14.3}$	43.0	162.4	63.1		166.3	77.7	1.26	9.26
$PEO^{19.7}$	46.3	166.2	63.4		169.8	79.3	1.26	9.46
$PEO^{28.8}$	41.6	134.7	61.7		139.8	65.3	1.26	7.78
$PEO^{45.9}$	39.8	136.1	61.6		143.0	66.8	1.26	7.96
$PEO^{69.2}$	40.3	126.1	63.1		128.4	60.0	1.26	7.15
PEO^{129}	38.4	122.3	64.4		120.6	56.4	1.26	6.73
PEO^{213}	40.8	117.9	64.2		119.9	56.0	1.26	6.67
PEO^{262}	41.3	121.8	64.7		117.3	54.8	1.26	6.53
PEO^{435}	39.5	120.6	66.3		117.5	54.9	1.26	6.55
PEO^{809}	41.8	119.6	65.6		114.5	53.5	1.26	6.38
PEO^{965}	42.4	123.1	66.1		120.7	56.4	1.26	6.73

3) SAXS data

To estimate the chain folding of polymer chains (Table S5), first, the length of the extended chain of PCL was calculated, considering the following equation:

$$L = nl_{\text{fiber}} \text{ (Eq. S1)}$$

where l_{fiber} is the length of two repeating units placed at the ideal intracrystalline chain conformation, i.e., 1.705 nm [1,2].

n is the number of such distances along the chain [3] and can be calculated via the following equation:

$$n = \frac{M_n - M_{chainends}}{2 \times M_{UR}} \text{ (Eq. S2)}$$

For the PCL samples listed in Table S5, all of which are synthetic, the total mass of the chain ends ($M_{chainends}$) was 107 g/mol. The term M_{UR} denotes the mass of the repeating unit in PCL. Finally, the number of folds per chain was then approximately estimated by:

$$\text{No. of folds per chain} = \frac{L}{l_c} \text{ (Eq. S3)}$$

where l_c is the lamellar thickness.

For PEO the number of folds has been estimated following the procedure reported in literature [4,5], in which average lamellar thickness, L, depends on the crystalline chain length, λ, and number of folds, n.

$$L = \frac{\lambda}{1+n}$$

The crystalline chain length is defined as follows,

$$\lambda = \frac{M_n}{\nu}$$

Being ν the molar mass per unit length along c axis, ν = 158.2 g/nm.

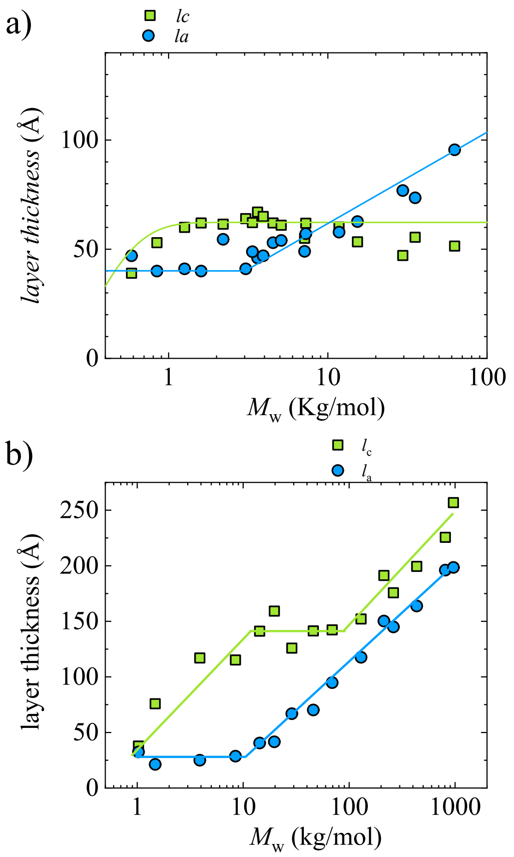

Figure S2. l_c and l_a vs. M_w for (a) PCL and (b) for PEO samples.

Table S5. Number of the folds per chain calculated by SAXS data for PCL and PEO samples.

Sample Name	M_n *(kg/mol)*	*n*	*Sample Name*	M_n *(kg/mol)*	*n*
$PCL^{0.58}$	0.48	0.7	$PEO^{0.19}$	0.194	
$PCL^{0.85}$	0.65	0.8	$PEO^{0.44}$	0.40	
$PCL^{1.26}$	0.96	1.1	$PEO^{0.62}$	0.58	
$PCL^{2.2}$	2	1.8	$PEO^{1.03}$	0.98	0.13
$PCL^{2.35}$	1.6	2.3	$PEO^{1.48}$	1.44	0.20
$PCL^{2.6}$	2.2	2.3	$PEO^{3.9}$	3.75	1.03
$PCL^{3.05}$	2.1	2.4	$PEO^{8.46}$	8.13	3.47
$PCL^{3.4}$	2.4	2.8	$PEO^{14.3}$	13.4	4.97
$PCL^{3.62}$	2.9	3.1	$PEO^{19.7}$	18.4	6.35
$PCL^{3.93}$	3.25	3.6	$PEO^{28.8}$	27.4	12.8
$PCL^{4.53}$	3.65	4.3	$PEO^{45.9}$	42.9	18.1

PCL$^{5.1}$	4.2	5.0	PEO$^{69.2}$	66.0	28.3
PCL$^{7.15}$	5.5	7.0	PEO^{129}	118.8	48.7
PCL$^{7.3}$	5.6	6.6	PEO^{213}	195.5	63.6
PCL$^{9.3}$	7.5	_	PEO^{262}	249.2	88.3
PCL$^{9.92}$	8.2	_	PEO^{435}	414	130.0
PCL$^{11.8}$	8.4	10.3	PEO^{809}	763.5	214.9
PCL$^{13.0}$	9.4	_	PEO^{965}	869	212.9
PCL$^{13.1}$	8.8	_			
PCL$^{15.4}$	9.6	13.3			
PCL$^{29.6}$	17.4	27.4			
PCL$^{35.4}$	22.1	29.6			
PCL$^{62.6}$	34.8	50.4			
PCL$^{126.9}$	70.5	107.4			

Number of folds and number of entanglements as a function of molar mass.

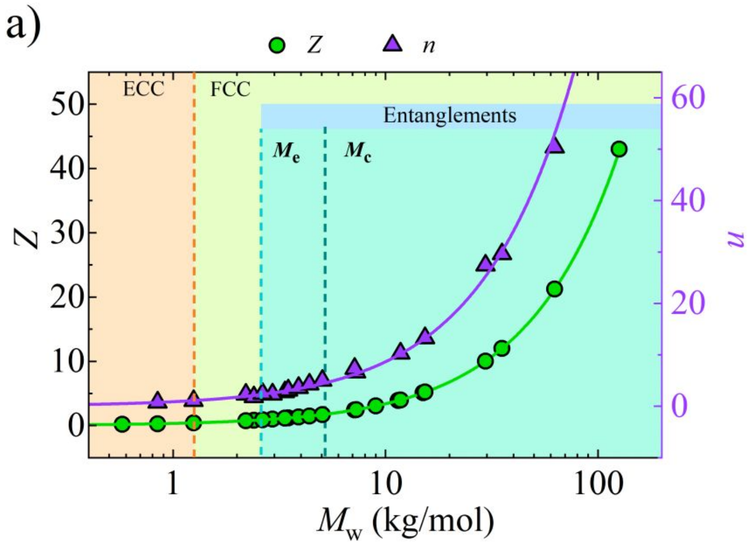

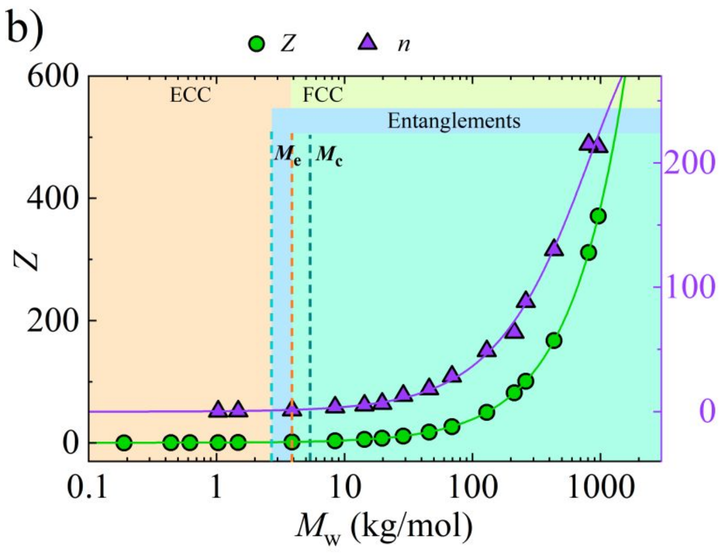

Figure S3. Number of entanglements (Z) and number of folds (n) for (a) PCL and (b) for PEO samples.

4) Self-nucleation (SN) measurements of ultra-high molecular weight polyethylene

The self-nucleation behavior of polyethylene was studied using the thermal procedure shown in Figure 5a. In this method, the material was heated to approximately 180 °C with a cooling and heating rate of 10 °C/min. Figure S3 summarizes the self-nucleation results: PE exhibits the three self-nucleation *Domains* but shows no melt memory. Above 131 °C, the sample is in *Domain I*, with an almost constant T_c value, despite some minor scattering. As T_s decreases, a narrow *Domain II* appears, designated as *Domain IIb*, since the DSC curve indicates the presence of unmolten crystals in the *DII* temperature range. Below 130 °C, the sample enters *Domain III*. The results clearly indicate a lack of melt memory, as there is no increase in T_c in the region where crystals are melted.

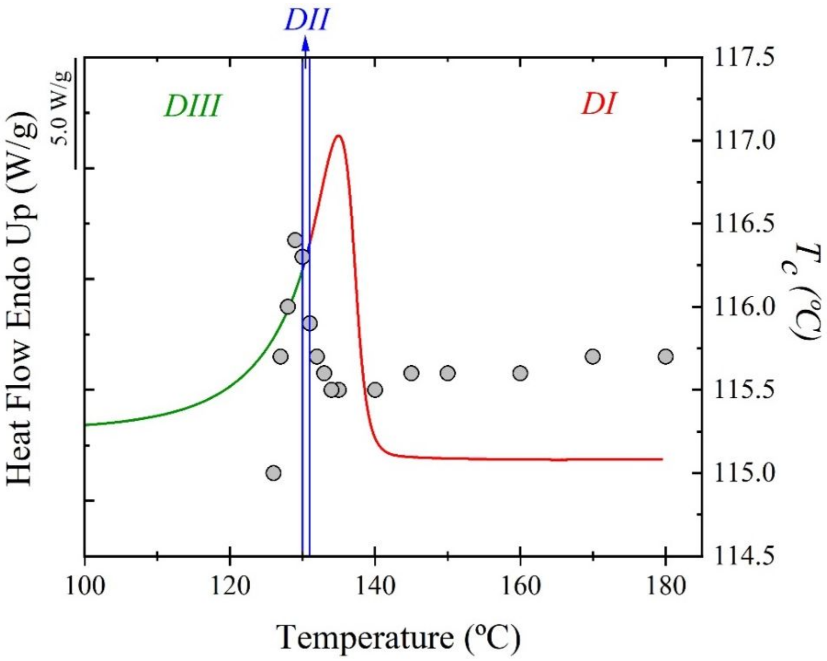

Figure S4. SN *Domains* for PE superposed on the standard DSC heating scan. T_cs are plotted on the right y-axis as a function of T_s. The DSC curves are color-coded to indicate the identified *Domains*. A single, very narrow *Domain II* (≈1 °C wide) is observed, with vertical blue lines marking the transition temperatures between *Domains*.

5) Calculation of the interaction index

To rationalize how intermolecular interactions and melt rheology control melt memory, we introduce a dimensionless interaction index defined as:

$$Interaction\ index = \frac{(\delta_p + \delta_h) \cdot X_c}{G^{0.5}} \qquad (Eq. S4)$$

where δ_p and δ_h are the Hansen polar and hydrogen-bonding solubility parameters (MPa$^{1/2}$), X_c is the experimental degree of crystallinity, and G is the shear modulus (MPa). As for what concerns the part of the index which takes into account directly intermolecular interactions, we did not consider the dispersion component (δ_d), as δ_d is essentially constant across PCL, PEO, and PE due to their similar methylene-dominated backbones, and therefore does not contribute significantly to differences in intermolecular interactions relevant to melt memory. This formulation is proportional to the effective density of intermolecular interactions within the crystalline regions (since it is proportional to the cohesive energy density) and, thus, should also correlate with their persistence in the melt.

We have employed the following values obtained by Abbott [6]:

PCL: δ_p (5 MPa$^{1/2}$) + δ_h (8.4 MPa$^{1/2}$) = 13.4 MPa$^{1/2}$

PEO: δ_p (10 MPa$^{1/2}$) + δ_h (5 MPa$^{1/2}$) = 15 MPa$^{1/2}$

PE: δ_p (0.8 MPa$^{1/2}$) + δ_h (2.8 MPa$^{1/2}$) = 3.6 MPa$^{1/2}$

6) Rheology

The master curves for PEO and PCL in Figure 2 were obtained by shifting horizontally and vertically the Small-Amplitude Oscillatory Shear (SAOS) data. The horizontal shift factors a_T are reported in Figure S4 (a) for PEO and Figure S4 (b) for PCL.

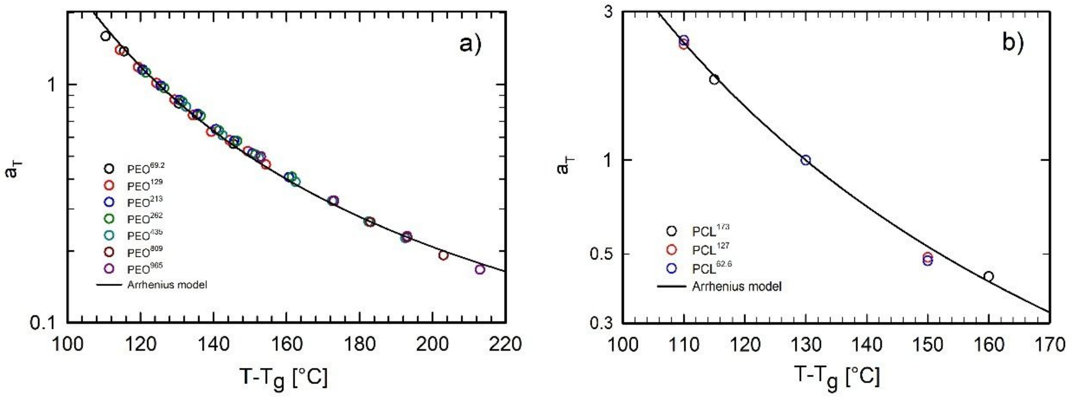

Figure S5. Horizontal shift factors for (a) PEO, at reference temperature T_g + 125 °C, and (b) PCL, at reference temperature T_g + 130 °C. The black continuous lines represent the Arrhenius model fit.

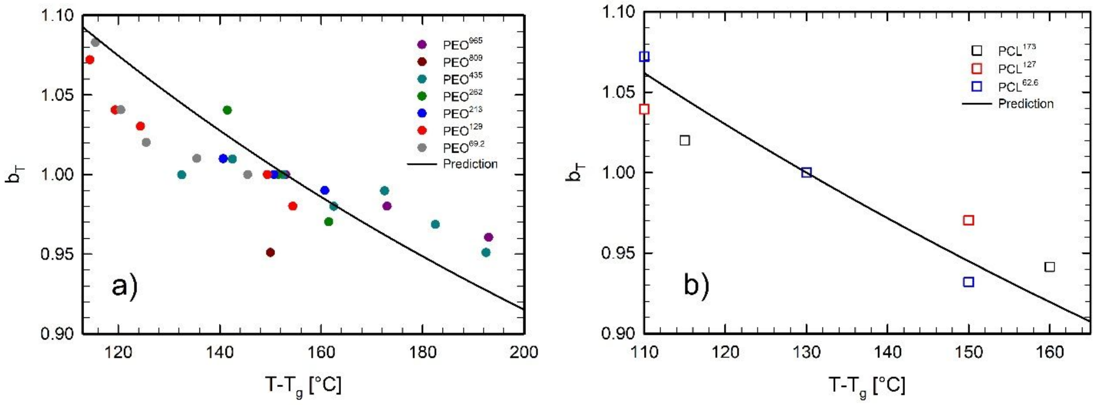

Figure S6. Vertical shift factors for (a) PEO, at reference temperature T_g + 125 °C, and (b) PCL, at reference temperature T_g + 130 °C. The black continuous lines represent the prediction $b_T = \rho(T_{Ref}) * T_{Ref} / (\rho * T)$.

At the same distance from the glass transition temperature T_g, the horizontal shift factors lay on the same curve given by the Arrhenius model, since all SAOS measurements were performed at temperatures well above T_g + 100K:

$$\log(a_T) = \frac{E_a}{R}\left(\frac{1}{T} - \frac{1}{T_{Ref}}\right),$$

where E_a is the activation energy, R the gas constant, T the absolute temperature and T_{Ref} the reference temperature chosen to shift the data. For PEO, the Arrhenius fit yielded E_a equal to 1.89 kJ/mol at $T_{Ref} = T_g + 125$ °C, and for PCL E_a equal to 2.24 kJ/mol at $T_{Ref} = T_g + 130$ °C. The vertical shift factors b_T are reported in Figure S5 (a) for PEO and S5 (b) for PCL, showing good agreement with the relation

$$b_T = \frac{\rho(T_{Ref}) * T_{Ref}}{\rho * T},$$

where ρ is the melt density at a given temperature T and $\rho(T_{Ref})$ is the melt density calculated at the reference temperature T_{Ref}.

References

[1] Y. Chatani, Y. Okita, H. Tadokoro, Y. Yamashita, Structural Studies of Polyesters . III . Crystal Structure of Poly-e-caprolactone, Polym. J. 1 (1970) 555–562.

[2] H. Hu, D.L. Dorset, Crystal Structure of Poly(iε-caprolactone), Macromolecules 23 (1990) 4604–4607.

[3] H. Su, H. Chen, A. Díaz, M. Teresa, J. Puiggalí, J.N. Hoskins, S.M. Grayson, R.A. Pérez, A.J. Müller, New insights on the crystallization and melting of cyclic PCL chains on the basis of a modi fi ed Thomson e Gibbs equation, Polymer (Guildf). 54 (2013) 846–859. https://doi.org/10.1016/j.polymer.2012.11.066.

[4] C. P. Buckley and A. J. Kovacs, Melting Behavior of low Molecular Weight Poly(ethylene-oxide) Fractions 2. Folded Chain Crystals, Colloid Polym. Sci., 1976, 254, 695–715.

[5] Zardalidis, G., Mars, J., Allgaier, J., Mezger, M., Richter, D., & Floudas, G. (2016). Influence of chain topology on polymer crystallization: poly (ethylene oxide)(PEO) rings vs. linear chains. Soft Matter, 12(39), 8124-8134.

[6] Abbott, S. Chemical Compatibility of Poly(Lactic Acid): A Practical Framework Using Hansen Solubility Parameters. In *Poly(Lactic Acid)*; John Wiley & Sons, Ltd, 2010; pp 83–95. https://doi.org/10.1002/9780470649848.ch7.
